# Thiol Reactive Probes and Chemosensors

**DOI:** 10.3390/s121115907

**Published:** 2012-11-19

**Authors:** Hanjing Peng, Weixuan Chen, Yunfeng Cheng, Lovemore Hakuna, Robert Strongin, Binghe Wang

**Affiliations:** 1 Department of Chemistry and the Center for Diagnostics and Therapeutics, Center for Biotechnology and Drug Design, Georgia State University, Atlanta, GA 30302, USA; E-Mails: hpeng2@gsu.edu (H.P.); wchen14@gsu.edu (W.C.); yfcheng@stanford.edu (Y.C.); 2 Department of Chemistry, Portland State University, Portland, OR 97207, USA; E-Mails: lhakun@pdx.edu (L.H.); strongin@pdx.edu (R.S.)

**Keywords:** thiols, cysteine, homocysteine, glutathione, hydrogen sulfide, sensors, probes, detection

## Abstract

Thiols are important molecules in the environment and in biological processes. Cysteine (Cys), homocysteine (Hcy), glutathione (GSH) and hydrogen sulfide (H_2_S) play critical roles in a variety of physiological and pathological processes. The selective detection of thiols using reaction-based probes and sensors is very important in basic research and in disease diagnosis. This review focuses on the design of fluorescent and colorimetric probes and sensors for thiol detection. Thiol detection methods include probes and labeling agents based on nucleophilic addition and substitution, Michael addition, disulfide bond or Se-N bond cleavage, metal-sulfur interactions and more. Probes for H_2_S are based on nucleophilic cyclization, reduction and metal sulfide formation. Thiol probe and chemosensor design strategies and mechanism of action are discussed in this review.

## 1. Introduction

**Figure 1.**
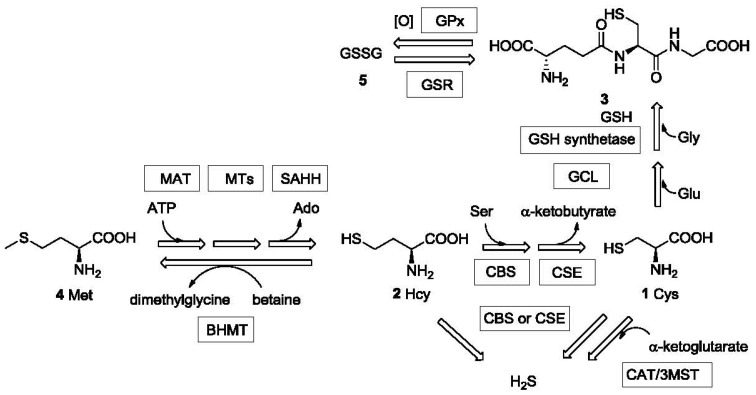
Metabolic relationship of Met, Cys, Hcy, GSH and H_2_S.

Sulfhydryl-containing compounds are often referred to as mercaptans due to their ability to react with mercury. They have unique chemical reactivity and thus special utility in chemical reactions [1] and in biological processes [2–4]. Thiols and thiophenols are widely used intermediates in synthetic chemistry; dithiothreitol (DTT) and 2-mercaptoethanol are common antioxidants used in biology labs [5]; aminothiols, such as cysteine (Cys, **1**), homocysteine (Hcy, **2**), and γ-L-glutamyl-L-cysteinylglycine (glutathione or GSH, **3**) play essential metabolic roles in biological systems. For example, Cys plays versatile roles in protein structure and function [6]. The sulfhydryl group of Cys serves as an ideal nucleophile in nucleophilic enzyme catalysis. Its ability to undergo reversible redox reactions under physiological conditions is essential for maintaining tertiary and quaternary protein structures through disulfide formation [7]. Hcy is a key intermediate generated during the biosynthesis of Cys from the essential amino acid methionine (Met, **4**) [3] (Figure 1). It is implicated in the health of the cardiovascular (CV) system [3]. The tripeptide GSH is present at very high levels (0.1–10 mM) in the cell (comprising about 90% of non-protein sulfur) and protects the cells against oxidative stress [2,8] among many other functions. GSH levels in cancer cells can impact the effectiveness of chemotherapy [9]. In addition to the above-mentioned thiols, thiol drugs such as D-penicillamine (D-PEN or PenA) [10] and tiopronin (TP or Thiola) [11], are also widely used in clinical practice. The quantitative detection of these drugs and their metabolites are very important in related clinical research. Hydrogen sulfide (H_2_S), the simplest mercaptan, has been known as an environmental hazard and toxic gas for many years. Recently, hydrogen sulfide has been recognized as one of the three gasotransmitters [12–15], together with nitric oxide (NO) and carbon monoxide (CO), that are endogenously produced and essential for maintaining the health of cardiovascular system among other roles. Even the unpleasant smell of mercaptans has found important applications. For example, some small molecule thiols such as ethanethiol and butanethiol are added to natural gas to help warn of gas leaks.

The metabolism and transportation of these sulfur-containing compounds in biological systems are closely related to a series of important enzymes and proteins, the deficiency of which could lead to various physiological/pathological conditions [16–18]. Furthermore, fluctuations in the endogenous concentration of these thiols indicate the functional state of the corresponding enzymes/proteins and are correlated with disease [18–20]. Thus, the detection of concentrations of mercaptans has implications and significance in clinical diagnosis. Among all the biologically important mercaptans, Hcy has been extensively studied as a biomarker for various reasons [21]. Deficiency in the expression of enzymes such as cystathionine β-synthase (CBS) and cystathionine γ-lyase (CSE or CGL) or their cofactors may lead to high levels or abnormal accumulation of Hcy, which characterizes inherited diseases such as homocystinuria [22], Down syndrome [23,24] and other clinical conditions such as vitamin (folate [25], cobalamin (vitamin B_12_) [26] or vitamin B_6_ [27]) deficiency, cardiovascular disease [28] and renal failure [29]. The tripeptide GSH is found to be present at low micromolar range in plasma [30]. However, the cytosol contains 0.1-10 mM GSH, depending on the cell type, while in most cells the concentration is 1-2 mM [4]. Because GSH provides antioxidant protection for the cell, the GSH concentration is also significant. The ratio of free GSH and its oxidized state glutathione disulfide (GSSG, **5**), which is normally >100:1 [31], is an indicator for both the corresponding enzyme (GSSG reductase or enzymes related to *de novo* GSH synthesis) activity and the redox state of the cell [32]. Low GSH concentration or [GSH]/[GSSG] ratio is related to inflammation and lung diseases, such as cystic fibrosis [19,33].

H_2_S is synthesized in the cell both enzymatically and non-enzymatically. The enzymatic synthesis of H_2_S is catalyzed by CBS, CSE [34,35] and cysteine aminotransferase (CAT)/3-mercaptopyruvate sulfurtransferase (3MST) [36,37]. H_2_S undergoes fast metabolism and is involved in the regulation of various systems, such as the cardiovascular [38–41] and the central nervous system (CNS) [42,43]. Concrete evidence has revealed the physiological and therapeutic significance of H_2_S, leading to a rapid growth in research activity involving H_2_S [13,14,44]. Endogenous and exogenous hydrogen sulfide has been demonstrated to exert either beneficial or detrimental effects in many pathological conditions. H_2_S was found to have therapeutic benefit in ischemia-induced heart failure [39,45] and hyperhomocysteinemia-induced hypertension [46]. The endogenous hydrogen sulfide level is related to Down syndrome [47] and lung diseases [48]. Exogenous hydrogen sulfide may confer myocardial protection against ischemia/reperfusion injury and exerts a protective effect against anti-inflammatory drug-induced gastric mucosal injury [38].

The utility of thiol detection is not limited to biomarker studies. Numerous kinetic assays have been developed based upon the quantification of thiols. These methods are used in studying enzymes that are naturally involved in the metabolism of thiols, such as CBS [49] and *S*-ribosylhomocysteinase (LuxS) [50], as well as enzymes that use artificial sulfur-containing substrates and can produce free thiols, such as acetylcholine [51], carboxypeptidase A [52], and so on.

From the above, one can clearly recognize the need for the development of chemosensors and probes for mercaptans, which has been reviewed in refs [53,54]. In the following sections, we present a variety of reaction-based chemoprobes reported for the selective detection of individual mercaptans, with a focus on strategies used for designing such chemoprobes. Due to page limitation, for applications of commonly used probes in biological samples such as plasma, urine and cell cultures, and detection procedures, readers are referred to recent reviews [21,55,56]. It should be noted that there are common challenges in the detection of mercaptans. They are easily oxidizable, and do not have readily detectable and distinguishable spectroscopic properties. Thus, the detection of thiols usually consists of reduction, chemical derivatization or labeling, and chromatographic separation followed by quantitative detection. Disulfides, such as GSSG, cystine, and homocystine, are commonly brought to their reduced state by the treatment of reducing agents such as DTT, tris(2-carboxyethyl)phosphine (TCEP) or NaBH_4_. In addition, selectivity among various thiols is a key issue, and methods for the accurate detection with minimal or no work-up, or *in vivo*, are the most desirable.

## 2. Probes for Biological Thiols

### 2.1. Labeling and Detection of Thiols Based on Nucleophilic Substitution

**Figure 2.**
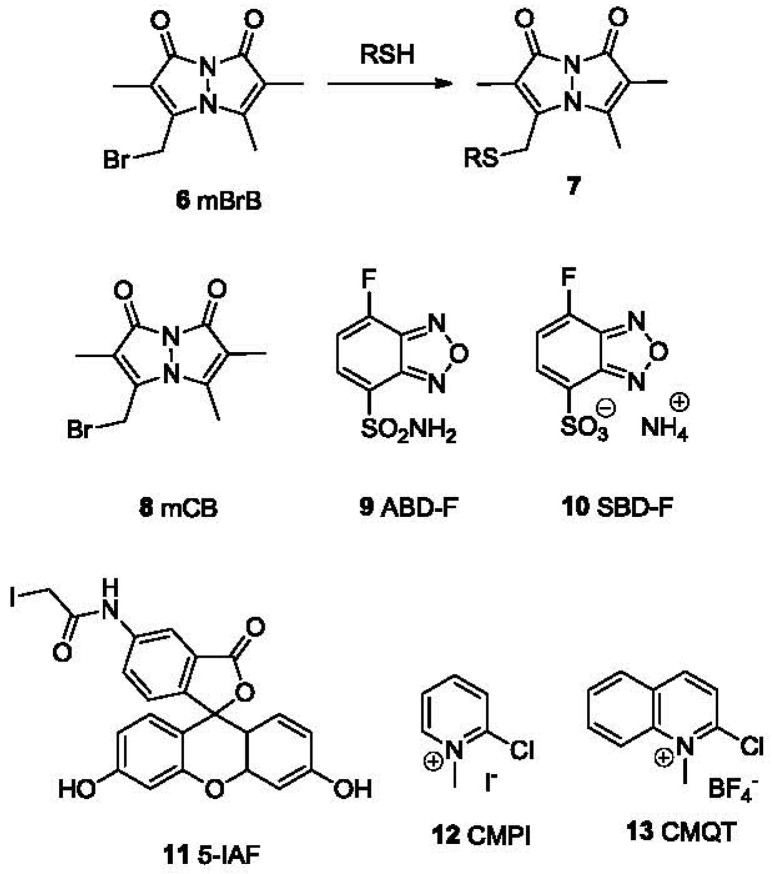
Thiol labeling agents bearing halogen groups.

Due to the strong nucleophilicity of the sulfhydryl group, it can react readily with electrophiles such as Michael acceptors and alkylating agents [1]. Therefore, some probes for thiols are either Michael acceptors or alkylating agents conjugated to a chromophore or fluorophore. The most widely used alkylating/labeling agents include monobromobimane (mBrB or mBB, **6**, Figure 2), 4-fluoro-7-sulfamoylbenzofurazan (ABD-F, **9**), 7-fluorobenzo-2-oxa-1,3-diazole-4-sulfonic acid ammonium salt (SBD-F, **10**), 5-iodoacetamidofluorescein (5-IAF, **11**), 2-chloro-1-methylpyridinium iodide (CMPI, **12**) and 2-chloro-1-methylquinolinium tetrafluoroborate (CMQT, **13**) (Figure 2). One common feature of these reagents is that they all bear a halogen that can undergo nucleophilic substitution with thiols. mBrB, ABD-F, SBD-F and 5-IAF form fluorescent conjugates with thiols, while CMPI and CMQT yield UV-absorbing conjugates. The samples could then be analyzed using high performance liquid chromatography (HPLC) or capillary electrophoresis (CE) coupled with a UV-vis detector (such as a diode array detector or DAD) or a fluorescence detector. Several commonly used fluorogenic and chromogenic labeling agents are compared in Table 1 in terms of their reactivity and limit of detection (LOD). Compared with absorption detection (such as CMPI and CMQT), fluorescent probes (such as mBrB) show higher sensitivity with detection limits in the picomolar range. This is due to the low background and less interference from the matrix. Compared to fluorescence detection, absorption methods, especially the absorptions in the UV range (<400 nm), are more prone to interference from other biological substrates. It should be noted that the detection limits listed in Table 1 were obtained from chromatographic methods, which includes an additional separation step. Such data are not directly comparable to the detection limits obtained from direct detections. In addition, detection limits reported for most probes by various labs were measured in different solvent systems (buffers or a mixed solvent of a buffer and an organic solvent). They are not directly comparable either.

Among these agents, mBrB (**6**) can easily undergo S_N_2 substitution with a sulfhydryl group at ambient temperature. The thiol-bimane conjugate **7** emits at 480 nm when excited at 380 nm. It has been extensively used in the quantification of thiols [19,57–60]. Automated separation-quantification of biological thiols in plasma and urine samples has been developed using mBrB [74]. Other bimane derivatives, such as monochlorobimane (mCB, **8**) [59], have also been used for thiol detection. ABD-F and SBD-F [75] consist another group of useful reagents for the derivatization of thiols, yielding fluorescent conjugates (λ_ex_ 380 nm, λ_em_ 510 nm) and have been used for detection of thiols in both plasma and tissues [62,65,76,77], although these reagents require fairly high temperature and pH for the substitution reaction. 5-IAF reacts rapidly with thiols at room temperature at pH 12.5 and is used in CE analysis of thiols in plasma and bacteria [66–68]. CMPI (**12**) forms thiol conjugates with an absorption maxima at 310 nm, providing nanomolar detection limits. It has been used for the analysis of thiols in urine and plasma [69,70]. CMQT (**13**) is user-friendly because it undergoes a fast substitution reaction with thiols. The conjugates show a maximum absorption at 355 nm. It has also been used for the quantitative analysis of thiols in urine and plasma [71–73,78,79]. Other UV labeling agents, such as *p-*bromophenacyl bromide, have also been reported [80]. However, the short absorption wavelength (263 nm) could limit their use because of spectral interference from the matrix.

These methods are widely used in both research and clinical analysis. However, one drawback of these agents is the lack of any selectivity among various thiols because they rely on simple nucleophilic addition or substitution for chromogenic or fluorescent labeling. As a result, detection and quantification of individual thiols using these techniques relies on separations such as HPLC or CE to differentiate various thiol-derivatives. Another disadvantage is interference from excess amount of labeling agent, especially in the case of fluorescence. This issue can be overcome if the labeling agent show significantly increased signal after conjugation.

**Figure 3.**
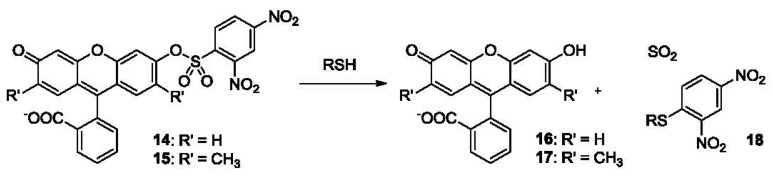
Fluorescent probes of thiols bearing a DNBS moiety.

Due to the high level of electron-deficiency on the phenyl ring, the 2,4-dinitrophenyl sulfonyl (DNBS) moiety can act as an electron sink when attached to a fluorophore and may incur photoinduced electron transfer (PET) resulting in the quenching of the fluorescence. Furthermore, DNBS can easily undergo de-sulfonylation in the presence of thiols, releasing SO_2_ gas and the attached fluorophore, thus resulting in a fluorescence increase (Figure 3). This strategy has been used in the development of many thiol probes. Examples include fluorescein derivatives **14** and **15** (Figure 3), which are almost non-fluorescent (Φ_FL_ 0.0007 and 0.0003, respectively in HEPES buffer at pH 7.4). In these two compounds, the hydroxyl group was “capped” as a sulfonate. After reaction with thiols in HEPES buffer, **16** and **17** are produced, leading to strong fluorescence (Φ_FL_ 0.75 and 0.58, λ_ex_ 460 nm, λ_em_ 560 nm). Compound **14** shows a low detection limit of 2 pM for thiols such as GSH and Cys. It was investigated for its application in cholinesterase (ChE) assays [81]. Although 0.7% of compound **14** was observed to be decomposed after 1 h incubation in buffer at 37 °C, high reaction rates with thiols still allowed for high throughput screening of ChE inhibitors using this probe.

**Figure 4.**
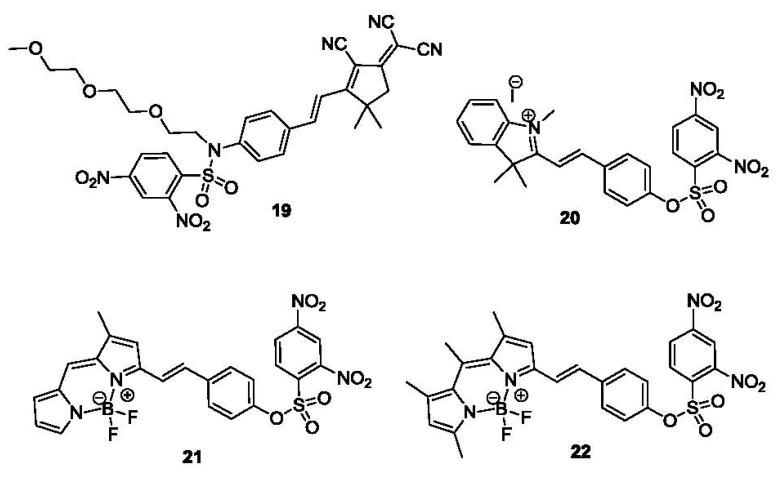
Fluorescent probes of thiols bearing a DNBS moiety.

In another example (compound **19**, Figure 4) the DNBS moiety was conjugated to a red-emissive fluorophore, which can be released through reaction with thiols [82]. A donor-π-acceptor architecture was built into the molecule, which was masked by the DNBS group. After addition of thiol, the sulfonyl ester moiety collapsed and resulted in the release of aniline as an electron donor. This process turned the fluorescence on with on/off ratios of 60 for GSH and 110-120 for Cys and Hcy (λ_ex_ 560 nm, λ_em_ 623 nm), providing a detection limit of about 3 μM. Almost exclusive selectivity was obtained for Cys over other amino acids. Furthermore, no hydrolysis was detected over 12 h at 37 °C. Its compatibility over a wide pH range (5.6–9.5) also makes this an excellent probe. The probe was utilized in the bioimaging of thiols in albino Swiss mouse embryonic fibroblast cells (3T3 lines). The probe showed good cell permeability and reacted with intracellular thiols. The control experiment using *N*-methylmaleimide to consume all free thiols did not show any fluorescence after incubation with the probe. In another example, a merocyanine fluorophore was conjugated to a DNBS moiety (Compound **20**, Figure 4) [83]. Cleavage of the DNBS moiety tunes the intramolecular charge transfer (ICT) state of the molecule and provides an absorption shift from 380 nm to 530 nm (ε∼78,000 M^−1^·cm^−1^) and a strong fluorescence emission at 553 nm. Both absorption and emission intensity are linearly related to thiol concentrations in MeOH/H_2_O 3:7. A nanomolar detection limit was achieved.

High photostability, high quantum yield and low pH sensitivity makes 4,4-difluoro-4-bora-3a,4a-diaza-s-indacene (BODIPY) one of the best fluorescent scaffolds available. Recently a BODIPY-based fluorescent probe (compound **21**, Figure 4) for thiols was reported [84]. The DNBS moiety was conjugated to this probe, providing an efficient fluorescence quenching effect. After exposure to Cys or GSH in PBS buffer for 10 min, a 20–25-fold increase in fluorescence (λ_ex_ 527 nm, λ_em_ 570 nm) was observed, presumably as a result of the displacement reaction described. Micromolar detection limit could be achieved with a moderate selectivity (>3-fold) over other amino acids. The probe was stable at pH 7.3 for at least 12 hr at room temperature. Imaging using this probe was demonstrated in monkey renal fibroblast COS-7 cell lines. In 2011, another BODIPY-based red-emitting off-on fluorescent probe (compound **22**, Figure 4) was reported [85]. This probe showed a 46-fold fluorescent enhancement after exposure to thiols in MeOH/H_2_O 4:1 (λ_ex_ 520 nm, λ_em_ 590 nm) with a detection limit of 7 μM. Probe **22** showed moderate specificity toward Cys over Hcy with about 3-fold difference, while no obvious response was observed for GSH. The fluorescence of the reaction product remained pH-independent over a wide pH range (pH 2–8). Cellular thiol imaging was performed in SGC-H446 cells. The specificity for thiols was also confirmed by *N*-methylmaleimide control experiments.

**Figure 5.**
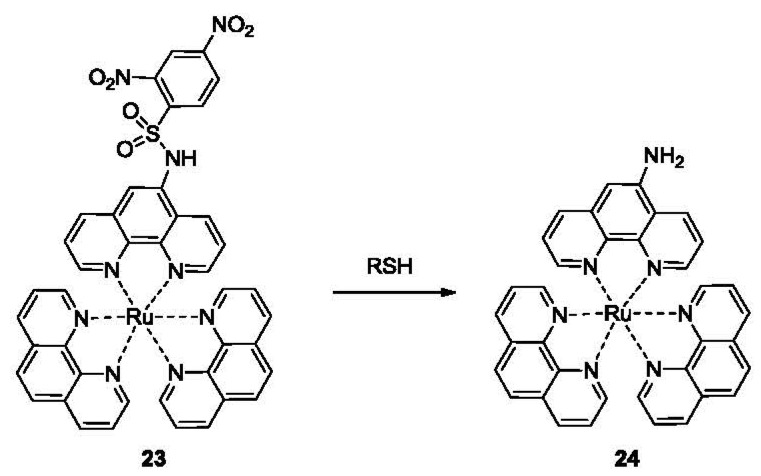
A phosphorescent probe of thiols bearing a DNBS moiety.

Compared to fluorescence, phosphorescence has the advantages of large Stokes shift, long luminescence lifetimes and ease of measurement. Phosphorescent probes utilizing a ruthenium complex have been developed for the detection of thiols based on the nucleophilic addition-fragmentation of DNBS (Figure 5, compound **23**) [86]. The probe **23** consists of a DNBS moiety attached to a Ru(II) poly(1,10-phenanthroline) complex as the luminophore, taking advantage of the metal-to-ligand charge transfer (MLCT) red emission. The addition of 20 μM of Cys led to the formation of **24** and a 90-fold increase in phosphorescence emission at 600 nm in a mixed solvent of acetonitrile/water 4:1 v/v, providing a detection limit in the high nanomolar range. The selectivity for Cys over other amino acids was over 40 fold. A fluorescent imaging study of this probe was performed using NCI-H446 cells. Control experiments were carried out with cells pre-treated with *N*-methylmaleimide, which covalently conjugate to thiols (this is also discussed in the next section).

### 2.2. Labeling of Thiols Based on Michael Addition

**Figure 6.**
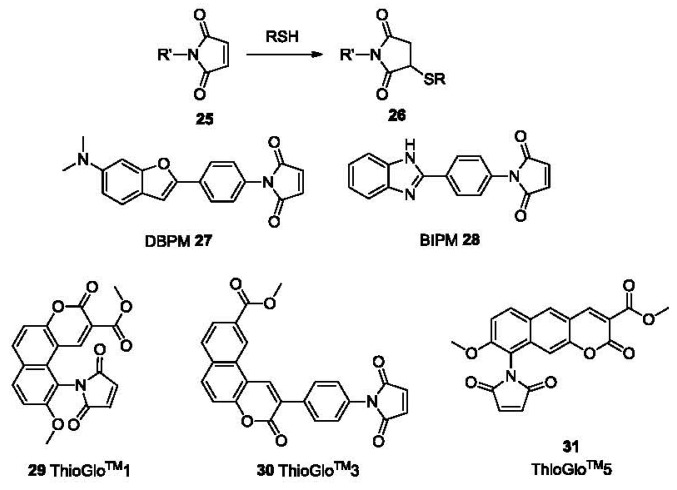
Labels for thiols using fluorescent maleimide conjugates.

Because of their excellent nucleophilicity, thiols react readily with Michael acceptors [1]. Recently, the Michael addition reaction has been widely used in the development of chemoprobes for thiols. Maleimide as one excellent Michael acceptor has found numerous applications in various studies. DBPM **27** and BIPM **28** (Figure 6) are such examples with off-on behaviors. Thus, maleimide has been conjugated to fluorophores of long wavelength leading to some commercially available fluorescent thiol-probes including ThioGlo™1 **29** (λ_ex_ 379 nm, λ_em_ 513 nm), ThioGlo™3 **30** (λ_ex_ 378 nm, λ_em_ 446 nm), and ThioGlo™5 **31** (λ_ex_ 365 nm, λ_em_ 536 nm). These probes react with thiols rapidly (usually in 2-5 min) and are used for fluorescent labeling of proteins. A HPLC-based study for the detection of thiols using ThioGlo™3 shows a detection limit as low as 50 fM for the derivative of GSH [87].

**Figure 7.**
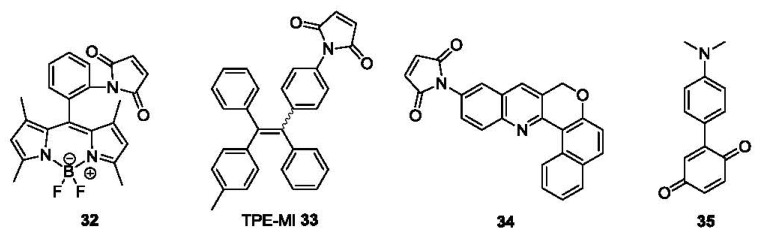
Michael addition-based probes.

A number of other probes bearing a maleimide group have been reported as fluorescent labels for thiols. In a recent example, a maleimide group was introduced at the *ortho* position of the phenyl ring of BODIPY derivative (**32**, Figure 7) [88], resulting in quenched fluorescence due to PET from BODIPY to maleimide. Experimental results showed that *ortho* substitution is crucial for the PET quenching. In terms of fluorescence quantum yields, they were 0.002 (*ortho*), 0.37 (*meta*) and 0.54 (*para*) respectively, which were determined using fluorescein (0.85) as the reference. After reaction with thiol, the PET effect was inhibited and the fluorescence of **32** was restored, affording a 350-fold intensity increase [89], acieving detection in the nanomolar range. Another example of a maleimide functionalized fluorescent probe is tetraphenylethene derivative **33** (TPE-MI, Figure 7). It showed an aggregation-induced emission (AIE) through thiol-maleimide addition in aqueous solution [90]. Though the selectivity among thiol containing molecules Cys, Hcy and GSH was low, the detection limit was as low as 1 ppb and the AIE occurred both in aqueous solution and the solid state.

Recently, another fluorescent probe for thiols using a Michael addition to the maleimide moiety was reported (Compound **34**, Figure 7) [91]. The detection limit of this probe was ∼20 nM for Cys based on S/N = 3. This probe was also evaluated in human metastatic breast cancer cells (MDA-MB 231) for imaging biothiols, using *N*-phenylmaleimide treated cells as a negative control.

Besides maleimide, the quinone moiety is also frequently chosen as a Michael acceptor for construction of thiol probes. For example, a donor-acceptor compound **35** was designed as a colorimetric probe (Figure 7) [92]. The probe showed an absorption band at 582 nm due to ICT. This band decreased upon addition of thiols such as Cys and GSH, mostly likely due to the nucleophilic addition of thiols on the quinone ring. A 17-fold fluorescence increase was observed for Cys in a 1:1 mixed solvent of H_2_O and THF The linear relationship between the absorption intensity change and the thiol concentration could be used for quantitation.

**Figure 8.**
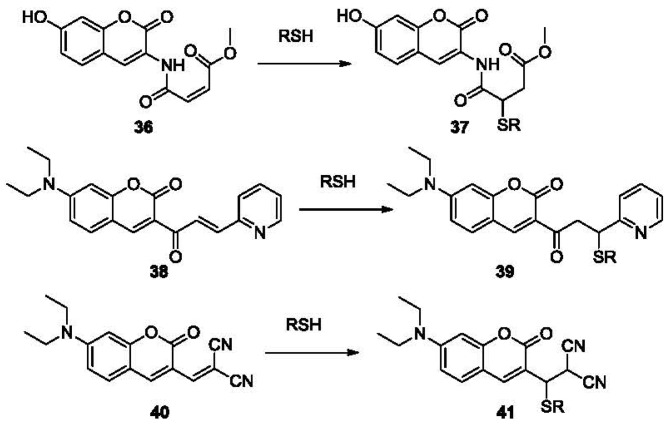
Fluorescent labeling of thiols using open chain Michael acceptors.

Open chain Michael acceptors are another option for the design of fluorescent sensor of thiols. A real-time thiol quantitation method reported was based on the modulation of intramolecular PET quenching upon addition of mercapto species [93]. Water soluble sensor **36** (Figure 8) reacts rapidly with thiols to form conjugate **37** with a rate constant of 7.0 × 10^4^ M^−1^·s^−1^ in Tris buffer at 25 °C. The reaction of 50 μM probe **36** and 50 mM β-mercaptoethiol has a t_1/2_ of 3 ms. The conjugate **37** (λ_ex_ 400 nm, λ_em_ 470 nm) has a fluorescence quantum yield more than 470-fold higher than that of **36**. The detection limit was as low as 0.5 nM. This has enabled the development of a high-throughput fluorescence assay for glutathione reductase, since the assay requires probes with very short response time. A similar idea was used in the design of α, β-unsaturated ketone derivative **38** (Figure 8) [94].

Upon thiol addition, the conjugation is disrupted and the fluorescence of the coumarin fluorophore is restored. The probe **38** is a highly sensitive thiol reagent showing over 200-fold increases in fluorescence (λ_ex_ 444 nm, λ_em_ 496 nm) by forming **39** through Michael addition. The detection limit was found to be 1 μM for Cys in 25 mM phosphate buffer (pH 7.4). The malononitrile group is an effective fluorescent quencher and has been utilized in another coumarin-based biothiol probe **40** (Figure 8) [95], which forms fluorescent product **41** through reaction with thiols. However compound **40** only showed a relatively low fluorescent enhancement upon reaction with thiols (5.6–12 fold, λ_ex_ 394 nm, λ_em_ 475 nm) in DMSO/HEPES buffer 1:2 (v/v).

**Figure 9.**
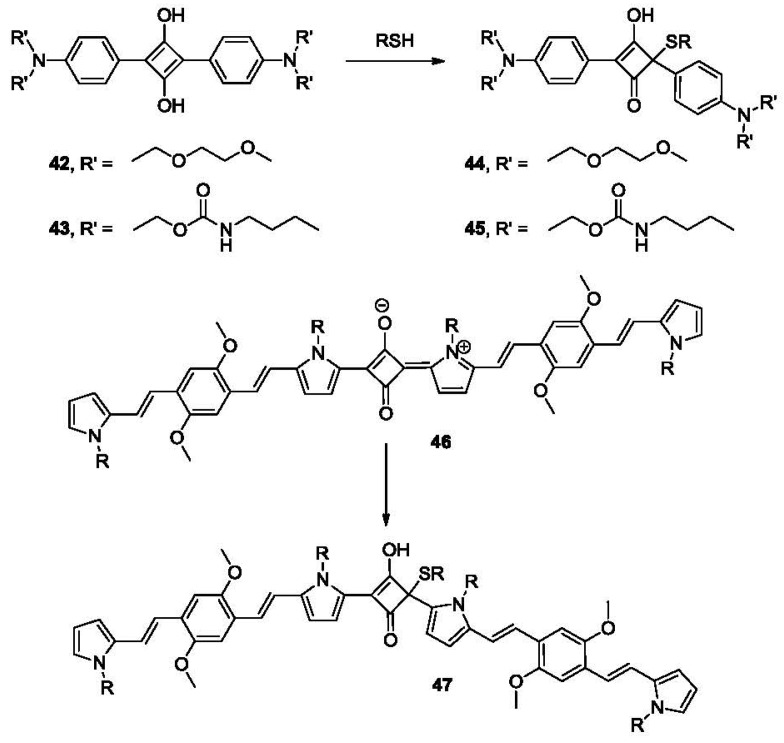
Fluorescent labeling based on the Michael addition using squaraines.

The nucleophilic addition of a sulfhydryl group to electron-deficient squaraines has also been used in the detection of thiols [96]. Compounds **42** and **43** are such derivatives (Figure 9). They showed strong absorption at 640 nm in acetonitrile/H_2_O. The addition of thiols to the solution of the probe in acetonitrile/MES buffer (pH 6.5) leads to the formation of adducts **44** and **45**, where the absorption band at 640 nm is significantly decreased, which is associated with a color change from blue to colorless. Near infrared (NIR) spectroscopy is emerging as a very powerful tool in tissue imaging because light in the 650–900 nm range is known to penetrate much deeper than visible light [97]. In 2009, a π-extended NIR squaraine dye **46** (Figure 9) formed by linking two bispyrrole molecules was reported [98]. Upon addition of thiols such as Cys and GSH, the π-conjugation of probe **46** is interrupted leading to the formation of **47**, which shows significantly increased fluorescence (λ_ex_ 410 nm, λ_em_ 595 nm and λ_ex_ 730 nm, λ_em_ 802 nm) in both visible and NIR region. The results also confirmed that the level of the aminothiols in blood doubles after smoking. Probes of similar structures have also been reported for cyanide detection [99]. However, since one would not normally expect cyanide in blood, the lack of selectivity over cyanide does not pose a significant interference problem.

**Figure 10.**
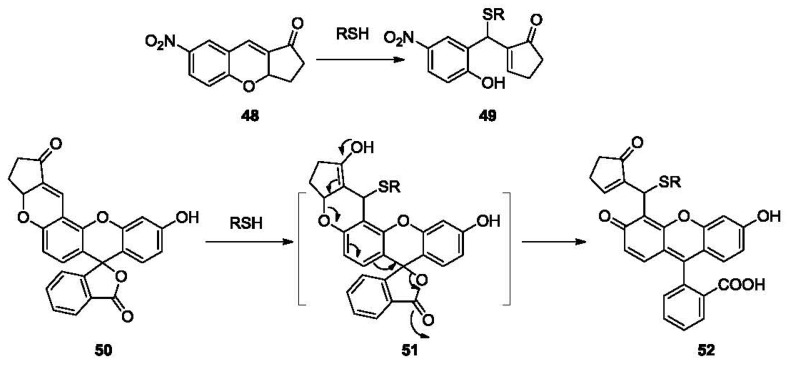
Fluorescent labeling of thiols based on Michael addition-ring opening.

Michael addition triggered ring-opening reaction is another strategy in developing thiol reactive probes (Figure 10). In 2009, a chromene-based colorimetric probe **48** (λ_max_ 292 nm) was reported [100]. The 4-nitrophenolate moiety was generated after Michael addition, leading to the formation of **49** (λ_max_ 405 nm) and a fast (within 10 s) visual color change from colorless to yellow. An analogous fluorescein-based probe **50** was reported for the detection of biothiol molecules [101]. Probe **50** responds to Cys, Hcy and GSH, forming fluorescent conjugate **52** through intermediate **51** with a detection limit in the high nM range. Further applications of the probe for intracellular thiol detection and monitoring of thiols in zebrafish were examined. The results showed that thiol species in the eye, gallbladder, egg and fin were detected by the probe after 5 μM 1 h exposure.

### 2.3. Detection of Thiols Based on Disulfide and Se-N Bond Cleavage

**Figure 11.**
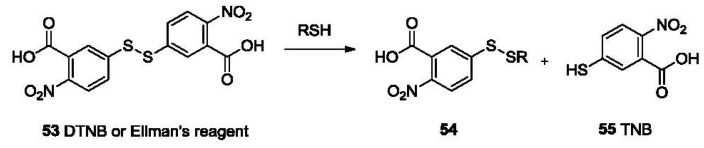
The reaction between Ellman's reagent and thiols.

Reducing ability is one of the most important properties of thiols. It imparts thiols with biological function and also enables the selective detection of mercaptans. Probes based on disulfide cleavage are commonly used for estimation of total sulfhydryl groups in protein samples. These probes, which usually exhibit weak absorption, share the structure of disulfide-linked chromophores. The reduction of the disulfide bond in the probe by sulfhydryl groups in the sample results in the production of a free chromophore, which absorbs strongly within the UV-vis range. The absorption could be recorded on a UV-vis spectrophotometer [102] or a multi-label counter [103]. One prominent example is the Ellman's reagent (5,5′-dithiobis-2-nitrobenzoic acid or DTNB, **53**, Figure 11), which was introduced in 1959 [104]. DTNB has two electron-deficient phenyl groups linked by a disulfide bond. This “activated” disulfide readily undergoes a transsulfuration reaction or reduction by a sulfhydryl group, releasing the conjugate **54** and the highly chromogenic product 5-thio-2-nitrobenzoate (TNB, **55**) with a strong absorption at 412 nm (ε = 14.15 × 10^3^ M^−1^cm^−1^ at 25 °C) [105].

**Figure 12.**
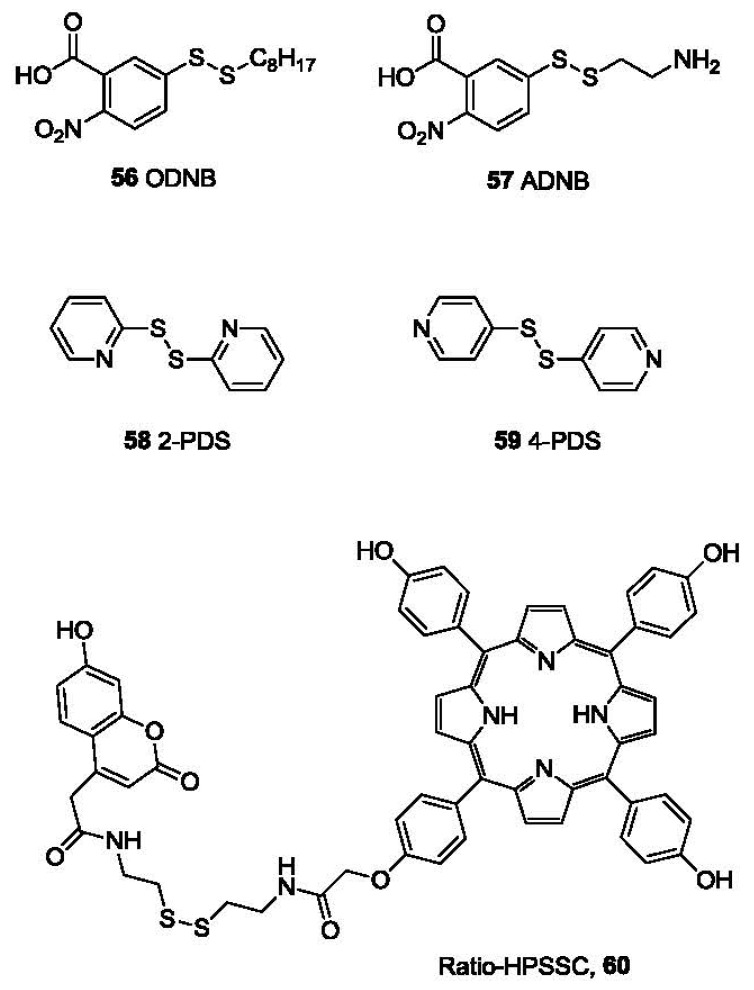
Colorimetric probes based on disulfide cleavage.

One problem with Ellman's reagent is its low stability. DTNB is relatively stable at pH below 8. Under more basic conditions DTNB undergoes obvious degradation, leading to undesired background absorption increase. However, slightly basic conditions (pH values greater than 8) are necessary for optimal reaction rates. Due to this reason, other reagents with relatively higher stability have been reported as alternatives. Such examples include *n*-octyldithionitrobenzoate (ODNB, **56**, Figure 12) [106] and 5-(2-aminoethyl)dithio-2-nitrobenzoate (ADNB, **57**) [107,108]. Both are mixed disulfides bearing one electron donating aliphatic chain. Because of the less activated disulfide bond and one less TNB group, they both show lower background than Ellman's reagent for thiol detection. ADNB was reported to show similar reactivity with thiols as Ellman's reagent with much slower hydrolysis [107,108]. On the other hand, the octyl group renders ODNB much more reactive to the sulfhydryl groups in proteins that reside in a hydrophobic environment. ADNB is more reactive to sulfhydryl groups in an anionic environment. These sulfhydryl groups usually react much slower with doubly negatively charged DTNB [106].

2,2′-Dipyridyl disulfide (2-PDS, **58**) and 4,4′-dipyridyl disulfide (4-PDS, **59**) [109,110] were reported for the determination of thiols such as GSH and protein thiols. When reduced by thiols, 2-PDS and 4-PDS forms 2-thiopyridone (2-TP) and 4-thiopyridone (4-TP), which absorb strongly at 343 and 324 nm, respectively. These two probes are reported to be more reactive to thiols at lower pH (3-6) compared with DTNB [111]. The protonation of the nitrogen on pyridinyl ring is believed to further activate the disulfide bond. Besides, 4-PDS reacts with GSH about 3 (pH 7)-30 (pH 4) times faster than 2-PDS [109].

Another interesting probe, Ratio-HPSSC (**60**) was developed by the Lin group [112]. In this probe (**60**, Figure 12), tetrakis(4-hydroxyphenyl)porphyrin is linked to coumarin by a disulfide bond. Due to the overlap of the coumarin emission (λ_ex_ 350 nm, λ_em_ 459 nm) with the porphyrin absorption (421 nm, Soret band) and subsequent Förster resonance energy transfer (FRET), the fluorescence of coumarin in Ratio-HPSSC is almost completely quenched. When exposed to thiols, the disulfide bond is cleaved, switching off FRET and thus restoring the fluorescence of coumarin. This probe shows good selectivity and sensitivity to thiols such as Cys with a detection limit lower than 1 μM in PBS buffer/ethanol. Thiol imaging in live Hela cells was also studied.

**Figure 13.**
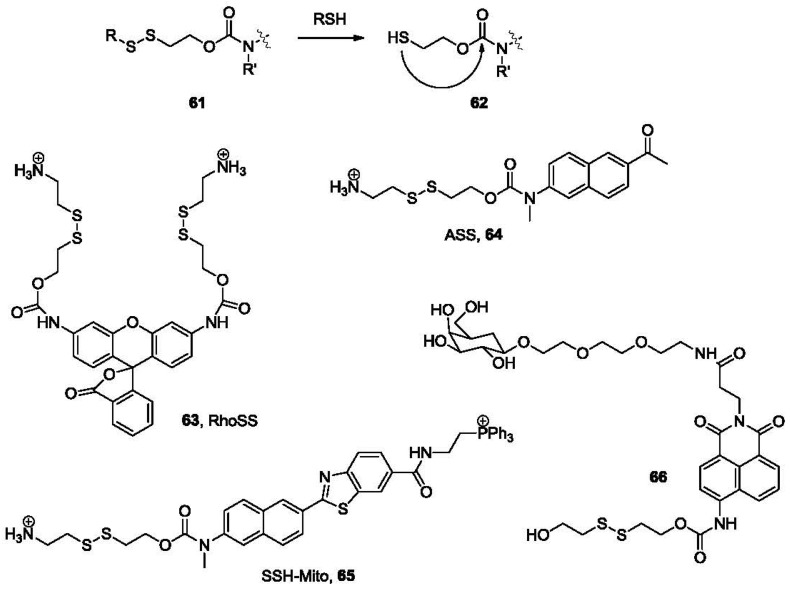
Fluorescent probes based on disulfide cleavage followed by cyclization release.

Cyclization-release is one of the most popular strategies used in directed drug release. This was also found to be useful in the development of thiol probes. In a typical example, a disulfide bond is linked to a fluorophore by a carbamate linkage (**61**, Figure 13). After the disulfide is reduced by thiols to form **62**, the newly formed sulfhydryl group could undergo an intramolecular cyclization with the carbonyl, releasing the linked fluorophore as a free amine. In the first example published in this class [113], **63** (RhoSS, Figure 13) released rhodamine 110 after incubation with thiols for 1–2 h at 37 °C resulting in a fluorescence increase at 535 nm. This probe was tested in cellular imaging studies in live Hela cells. Strong fluorescence response was observed after incubating with cells. When incubated with *N*-ethylmaleimide pre-treated cells, where sulfhydryl groups on thiols are capped, no such fluorescence response was observed. The cellular distribution of the probe was studied by co-staining and the probe seems to be localized within the cytosol.

Two-photon microscopy (TPM) [114,115] is a relatively new technology that has received great interest in the past several years owing to its applications in deep tissue imaging (>500 μm). Specifically, a two-photon microscope generates pulsed laser beams, focusing within less than femtoliter volumes in the objective, and employs two photons of lower energy to excite the fluorophore. TPM allows for increased penetration depth, localized excitation, reduced photo-damage and prolonged observation time [116,117], and thus is superior to conventional one-photon excitation and confocal microscopy. Recently, two-photon fluorescent probes that could selectively image biothiols have been developed. For example, ASS (**64**, Figure 13) is a two-photon fluorescent probe derived from 2-methylamino-6-acetylnaphthalene. In the presence of thiols the disulfide bond is cleaved, leading to intramolecular cyclization and release of the fluorophore, which could be detected by TPM. Fluorescence imaging using this probe in live cells and rat tissue in a depth of 120 μm has been demonstrated [118]. Based on an analogous concept, SSH-Mito (**65**, Figure 13) bearing a triphenylphosphonium (TPP) moiety for mitochondrial targeting was developed recently [119]. TPP has been demonstrated to specifically transport cargo molecules to mitochondria due to electrostatic interactions of the positively charged phosphine and the negative potential across the inner mitochondrial membrane [120].

Recently, a thiol probe aimed at targeting liver cells was described [121]. This probe (**66**, Figure 13) contains a galactose subunit and a disulfide-linked naphthalimide. The terminal galactose residue is recognized by the asialoglycoprotein receptor (ASGP-R), which is expressed on the plasma membrane of mammalian hepatocytes. This directs the probe selectively to hepatocytes. In the presence of biothiols, such as GSH, thioredoxin (Trx), Hcy and Cys in PBS buffer, the disulfide bond is cleaved, followed by an intramolecular cyclization. This uncaps the amino group on the naphthalimide moiety and leads to a substantial increase in fluorescence intensity at 540 nm. A 10-fold fluorescence increase was observed for thiols at 5.0 mM when 1.0 μM of probe **66** was used in PBS buffer. The target specificity was confirmed by cellular imaging experiments, in which fluorescence was observed only in HepG2 cells, but not in other non-hepatocytes such as C2C12, HaCaT, and N2a cells. The results of tissue imaging experiments using male Sprague-Dawley rats have also confirmed the specificity.

**Figure 14.**
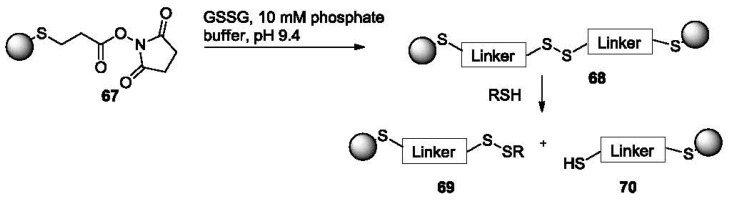
Detection of thiols using disulfide linked AuNPs.

Probes for the detection of thiols based on gold nanoparticles (AuNPs) have also been reported [122]. Specifically, disulfide linked AuNP clusters (**68**, Figure 14) are formed by treating AuNPs coated with dithiobis(succinimidylpropinate) (DSP, **67**).

The formation of AuNP clusters was confirmed by localized surface plasmon resonance (LSPR) spectroscopy and transmission electron microscopy (TEM). When the AuNP clusters are exposed to small molecule thiols, the GSSG disulfide linkage can be readily reduced, resulting in the reversal of the cluster formation to form **69** and **70**. This is characterized by a significant blue shift (610 nm to 520 nm) of the LSPR spectra. This probe is especially useful for the detection of low molecular weight thiols because by controlling the size of the linker and the NPs, a steric environment is created for the easy access of low molecular weight thiols but not larger molecules. Different responses were obtained for various thiols with the best detection limit (to NaSH) being in the low micromolar range.

**Figure 15.**
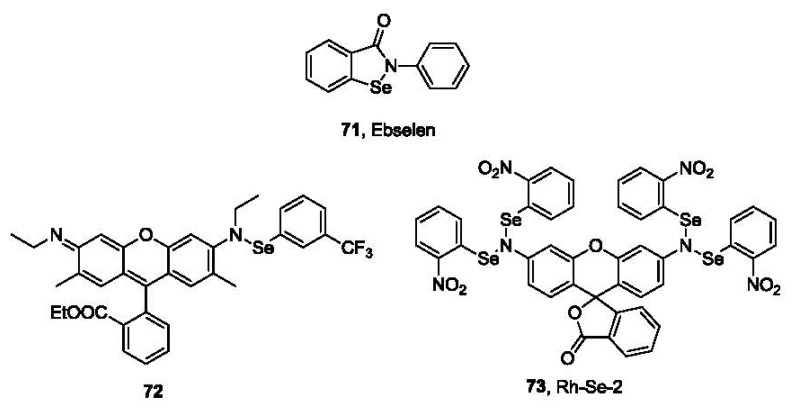
Ebselen and probes based on Se-N bond cleavage.

Glutathione peroxidase (GPx) is an antioxidant enzyme that catalyzes the oxidation of GSH to GSSG by H_2_O_2_. It has a selenocysteine in its active site, forming a catalytic triad with tryptophan (Trp) and glutamine (Gln). The catalytic mechanism involves the formation of selenyl sulfide as an intermediate [123]. An anti-inflammatory drug Ebselen (**71**, 2-phenyl-1,2-benzisoselenazol-3(2H)-one, PZ 51 or DR3305, Figure 15) [124–126], bearing a Se-N bond, could also catalyze the reduction of H_2_O_2_ by GSH with a similar mechanism. Based on this well-known reaction between thiols and the Se-N bond, Tang and co-workers developed a fluorescent probe for thiols [127]. Probe **72** is only weakly fluorescent in aqueous solution. After incubation at 25 °C for 30 min with GSH, the Se-N bond is cleaved, leading to the formation of rhodamine 6G and the concomitant significant increase of fluorescence (λ_ex_ 525 nm, λ_em_ 550 nm). The reaction is fast and quantitative with a linear correlation curve between fluorescence intensity and thiol concentrations. The detection limit is lower than 100 nM in PBS buffer. This probe was used to sense the difference in thiol concentrations in normal human liver cell line HL-7702 and human hepatoma cell line HepG2.

Another example based on Se-N bond cleavage has also been reported [128]. This probe (**73**, Rh-Se-2, Figure 15) reacts with thiols at 25 °C in PBS buffer with the maximum fluorescence at 522 nm observed after only 5-10 min. This method also provides a linear calibration curve with a detection limit lower than 100 nM. Cellular imaging using this probe has also been tested in HL-7702 and HepG2 cells.

### 2.4. Sensors Showing Selectivity among Thiols

Almost all the labeling and detection methods described thus far are based on the reactivity of the sulfhydryl group itself, which is present in all thiols, such as Cys, Hcy, GSH, and Cys residues in proteins. Therefore, there is no reason to expect more than limited selectivity among various thiols for idiosynchratic reasons. However, considering the difference in the biological roles of each thiol, the quantification of individual thiols instead of total thiols, is very important. For example, total Hcy concentration (tHcy) in healthy plasma is lower than 15 μM [21], while the Cys concentration is 20–30 times that of tHcy. Because tHcy is related to many diseases, its selective determination is necessary. Differentiation among various thiols in current methods is mainly based on chromatographic separation [56,78,129]. Specifically, thiols are derivatized with labeling agents such as mBrB or CMQT and analyzed by HPLC or CE equipped with a DAD or fluorescence detector. Data could then be compared with a calibration curve. This process is instrument-dependent and time-consuming. Therefore, the need for the development of chemoprobes, which allow for selectivity among thiols, is urgent.

**Figure 16.**
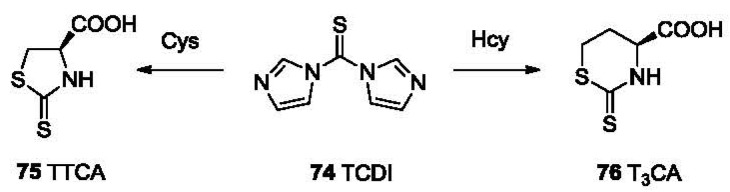
TCDI in the detection of Cys and Hcy.

1,1′-Thiocarbonyldiimidazole (TCDI, **74**, Figure 16) [130,131] has been reported to differentiate Cys, Hcy and PenA from other non-aminothiols. Through dual nucleophilic substitution steps accompanied by releasing two molecules of imidazole, TCDI selectively reacts with aminothiols such as Cys, Hcy, PenA and cycteinylglycine. The five-membered ring adduct (**75**, TTCA) formed from the reaction with Cys has a maximum UV absorption at 272 nm. The six-membered ring adduct T_3_CA (**76**) formed from Hcy absorbs at 283 nm. The reaction completes within 20 min at 37 °C under basic aqueous conditions. HPLC can then be used to quantitatively analyze those adducts.

**Figure 17.**
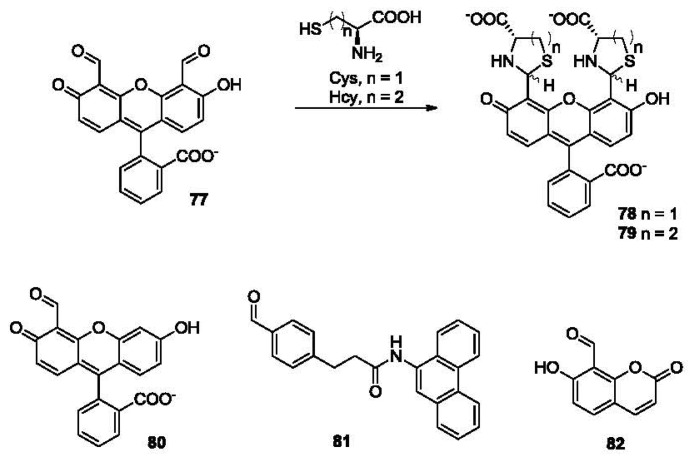
Fluorescent probes bearing an aldehyde group.

In 2003, the Strongin group reported the synthesis and evaluation of a bisaldehyde based fluorescent sensor **77** (Figure 17) for thiol detection [132]. The formation of a thiazolidine (**78**) between aldehyde and Cys led to a 25 nm red shift in the absorption spectrum (from 480 to 505 nm) and a decrease in fluorescence intensity.

This sensor responds to Hcy as well, giving rise to a six-membered ring product **79** with very similar spectral changes (about 25 nm of red shift in the absorption spectrum and a decrease in fluorescence intensity). The same group has also synthesized the monoaldehyde **80** [133]. Interestingly, the results have shown that both sensors exhibited moderate selectivity to Cys over Hcy. This might be due to the favored formation of a five-membered ring structure. In 2004, the Barbas group reported another probe **81** bearing an aldehyde group [134]. This probe showed a fluorescence increase after condensation with Cys. Although the authors did not mention the study of homocysteine, this sensor showed a significantly higher reaction rate with Cys compared to GSH. Based on the same design strategy, the Hong group described in 2008 a fluorescent probe **82** bearing an aldehyde moiety on the coumarin dye. This probe was reported to show about 3-fold selectivity toward Cys over Hcy with no response to GSH [135]. In addition, two photon fluorescent probes have been reported recently bearing an aldehyde moiety, showing selectivity for cysteine [136].

**Figure 18.**
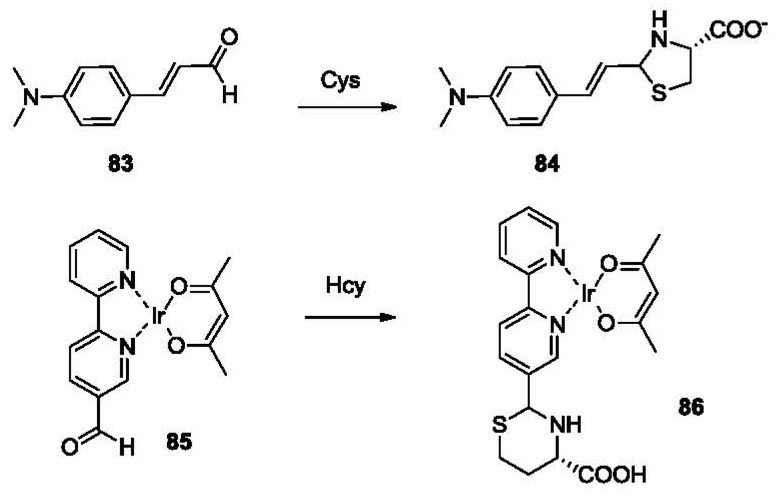
Selective detection of Cys and Hcy using aldehyde-bearing probes.

In 2005, the Strongin group reported the selective detection of Cys using a commercially available aldehyde (Figure 18, **83**) [133]. This probe shows exclusive selectivity to Cys over Hcy, forming an adduct **84**, which is demonstrated by a decrease in absorbance at 400 nm. The addition of Hcy did not lead to any obvious change in absorption. This Cys-selective sensor is very useful, because it could be used to determine background concentration of Cys in the biological sample. Then the concentration of Hcy may be calculated by subtracting the concentration of Cys from the total concentration of biological amino thiols, which normally includes Cys and Hcy. One way of achieving such selective detection is to use excessive amount of **83** to selectively pre-saturate the Cys in the sample. This is then followed by a direct detection of Hcy using probe **77**. This turned out to be a very effective method. After pre-treatment of **84**, the absorption decrease of **77** was only due to the concentration of Hcy.

In 2007, a phosphorescent probe was reported for the detection of biothiols. A homocysteine-selective iridium(III)-based sensor **85** was synthesized [137]. This probe reacts with Cys to form **86**, showing a color change from orange to green and an increase in phosphorescence (λ_ex_ 360 nm, λ_em_ 525 nm) in DMSO/HEPES buffer. It also exhibited a 19-fold selectivity for Hcy over Cys. This was the first luminescent chemosensor with high selectivity for Hcy over other biothiols (including Cys and GSH).

**Figure 19.**
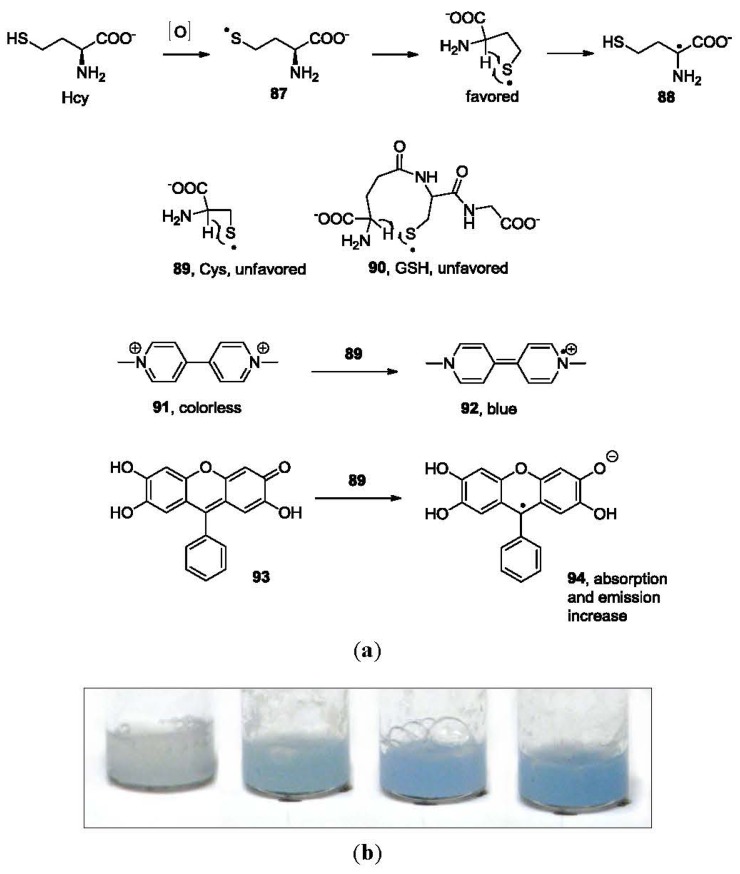
(**a**) Selective detection of Hcy based on electron transfer from a carbon-centered radical on Hcy, (**b**) Hcy serum calibration standards after treatment with **91**; from left to right: 3.79, 6.13, 13.4 and 38.73 μM respectively.

In addition to the sensors based on aldehydes, another approach for selective detection of Hcy was described by the Strongin group [133,138]. Thiyl radicals (**87**, **89** and **90**, Figure 19) are the intermediates in the oxidation of thiols such as Cys, Hcy and GSH. They can either lead to disulfide formation, or a “repairing” process, which results in the formation of a carbon centered radical (**88**). The intramolecular formation of the carbon centered radical is favored for Hcy, because it goes through a 5-membered ring transition state, and is less favored for Cys and GSH. In the presence of a compound that develops a colored radical species, the carbon-centered radical can be detected. Methyl Viologen (**91**) is a good candidate. After gently refluxing for 5 min, the mixture of **91** and Hcy in Tris buffer showed a blue color through formation of **92**, demonstrated by absorption at 398 and 605 nm. The same response was not observed for either Cys or GSH. This method was also applied to the selective detection of Hcy in human plasma [139]. Figure 19(b) shows an optimization of the method for total homocysteine in human serum standard calibrators using **91** (unpublished results). Fluorone black (**93**) can also be used for Hcy detection using this mechanism. This is demonstrated by an absorption increase at 510 nm and emission increase at 540 nm in 70% MeOH in H_2_O.

**Figure 20.**
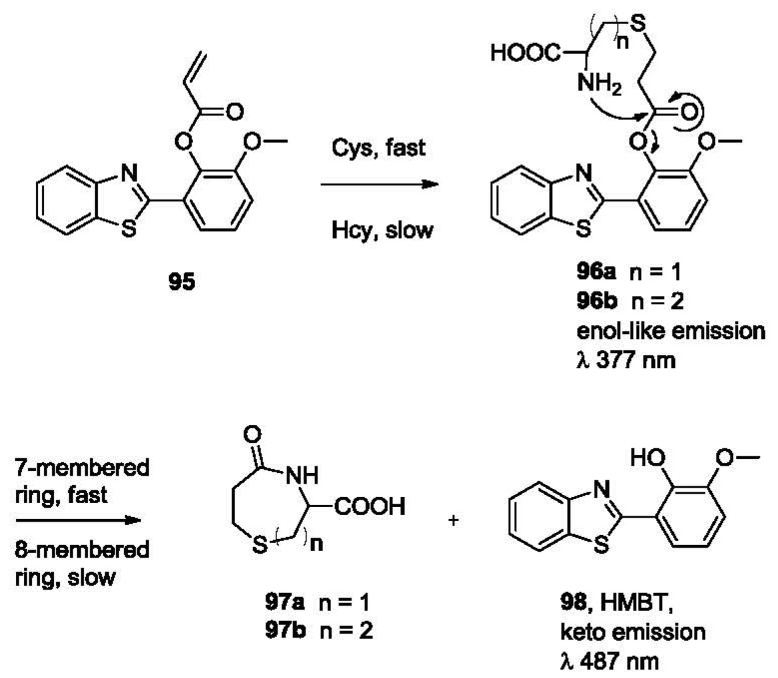
Discrimination of Cys and Hcy based on Michael addition-lactam formation cascade.

The most recent work on the selective detection of Hcy and Cys reported by the Strongin group takes advantage of a Michael addition-lactam formation cascade [140]. The probe (**95**, Figure 20) undergoes a Michael addition with the sulfhydryl group to generate conjugates **96**. Then an intramolecular nucleophilic attack from the amino group leads to the formation of lactams **97**, release of a HMBT fluorophore **98**, and a subsequent fluorescence change. The difference in reaction rates with Cys and Hcy provides an option for discrimination of Cys and Hcy. Very recently, Yoon lab has also reported a cyanine-based NIR emitting fluorescent probe showing selectivity for cysteine using the same strategy [141].

## 3. Probes for Aromatic Thiols

**Figure 21.**
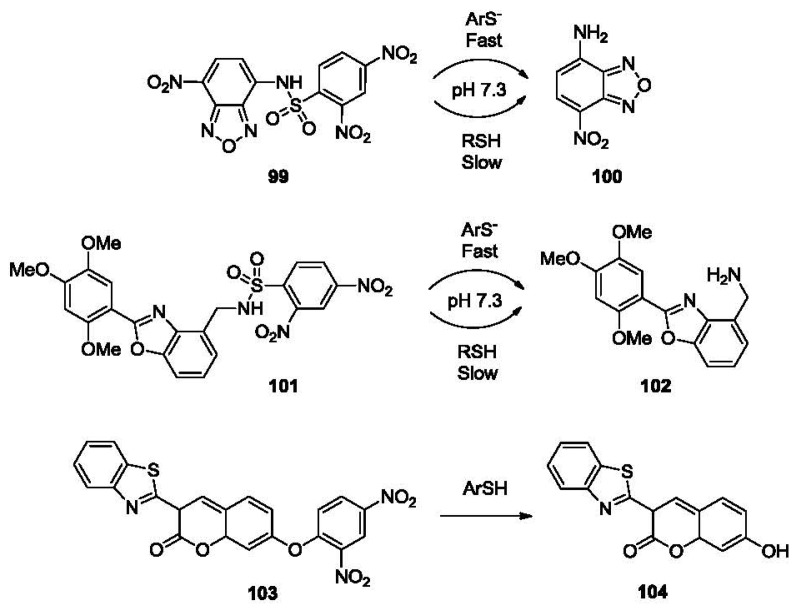
Fluorescent probes of thiophenols.

Aromatic thiols, such as thiophenols, are widely used in the production of pharmaceutical intermediates, polymers and pesticides [142]. Exposure to thiophenol may lead to damage to the central nervous system, kidney and liver [143]. Due to conjugation effect with the phenyl ring, the sulfhydryl group of thiophenol has a pK_a_ much lower than that of alkyl thiols. Thus it is conceivable that fluorescent labeling agents for thiols would also label thiophenols, and probably more efficiently. The significant difference in the nucleophilicity under physiological pH conditions has been successfully used in the selective detection of thiophenols. The Wang group has reported two fluorescent chemoprobes for thiophenols [144,145]. Compound **99** is based on a nitrobenzofuranzan fluorophore bearing a DNBS moiety (Figure 21), which shows no fluorescence before the addition of thiophenol. When thiophenol is added, the sulfhydryl group attacks the dinitrophenyl and releases the free fluorophore NBD (**100**), which leads to a dramatic fluorescence increase at an emission wavelength of 550 nm. A similar design also imparted the same selectivity to compound **101**, which releases a benzoxazole fluorophone **102** after the addition of thiophenol. Both probes have a detection limit in the low micromolar range.

Besides the strategy of using DNBS moiety, another probe (**103**, Figure 21, Φ_FL_ 0.006) based on the nucleophilic attack on the dinitrophenyl group was developed by Lin and co-workers [146]. The probe releases a fluorescent coumarin derivative **104** (λ_ex_ 461 nm, λ_em_ 494 nm, Φ_FL_ 0.50) upon reaction with thiophenol, showing good selectivity for thiolphenol over 2-mercaptoethanol. The detection limit was reported to be 1.8 nM for benzenethiols. This method has been tested in water, soil and cell cultures.

## 4. Probes for H_2_S

H_2_S has been recognized as an endogenously produced gasotransmitter. It plays regulatory roles in multiple systems, such as the cardiovascular [38–41] and the central nervous system (CNS) [42,43]. It also shows therapeutic effects in heart diseases [39,45]. Due to the newly recognized biological significance of H_2_S, more and more research interest has been focused on the molecular mechanisms of its biological functions and related therapeutic applications. However, H_2_S is very unstable because of its high volatility and high propensity to be oxidized under physiological conditions. Such properties have made the accurate measurement of hydrogen sulfide a difficult task. A number of methods have been reported for the detection of H_2_S [147,148]. These methods include gas chromatography (GC) [149–152], HPLC [153], and electrochemical methods, which mostly rely on sulfide ion selective electrodes [154,155] or polarographic methods [156]. Despite the availability of numerous detection methods, literature reported hydrogen sulfide concentrations vary significantly among publications, ranging from high micromolar [48,157,158] to low nanomolar [159]. Part of the reason could be the difference in sample preparation and intrinsic fluctuation of hydrogen sulfide concentrations. Other reasons might be due to the lack of methods for fast and selective detection. In any case, it is generally agreed that the development of new approaches is necessary not only for the selective and instantaneous detection, but also for intracellular imaging.

### 4.1. Probes for H_2_S based on Nucleophilic Cyclization Reactions

**Figure 22.**
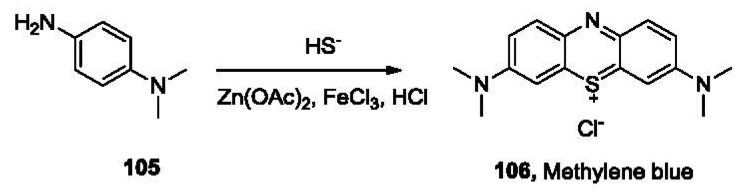
The reaction involved in H_2_S detection using the methylene blue method.

Hydrogen sulfide (H_2_S) dissociates in aqueous solutions in two sequential steps to HS^−^ and S^2−^ with a pK_a1_ of 6.9 and pK_a2_ of 12, which means over 75% of H_2_S exists in the anionic state at physiological pH. Sulfide is a stronger nucleophile than commonly encountered anions such as chloride and hydroxide under physiological conditions. This provides the possibility of selective detection of sulfide amongst various anions. One of the classical methods for the quantification of sulfide is the methylene blue method [160]. Sulfide reacts with *N,N-*dimethyl-*p*-phenylenediamine (**105**, Figure 22) in the presence of Zn(OAc)_2_ and FeCl_3_ under acidic conditions, yielding methylene blue (**106**), which absorbs strongly at 670 nm.

Samples containing H_2_S are often preserved by addition of Zn^2+^ to trap H_2_S in the form of ZnS. During analysis, solutions of compound **105** and FeCl_3_ in HCl are added into the sample. A blue color, which could be measured by a UV-vis spectrophotometer, usually develops in minutes [161]. This method shows both good selectivity and sensitivity with a nanomolar detection limit. It has been approved by the US Environmental Protection Agency (EPA) as a standard method for sulfide quantitation and has been utilized for hydrogen sulfide determination in many studies [153,157,162]. However, because methylene blue tends to form dimers and trimers [163] at concentrations over 10 μM, a linear calibration curve could not be obtained for sulfide at 10 μM or higher concentrations. In addition, the use of corrosive reagents and the non-instantaneous nature of this method have also limited the application of this method.

**Figure 23.**
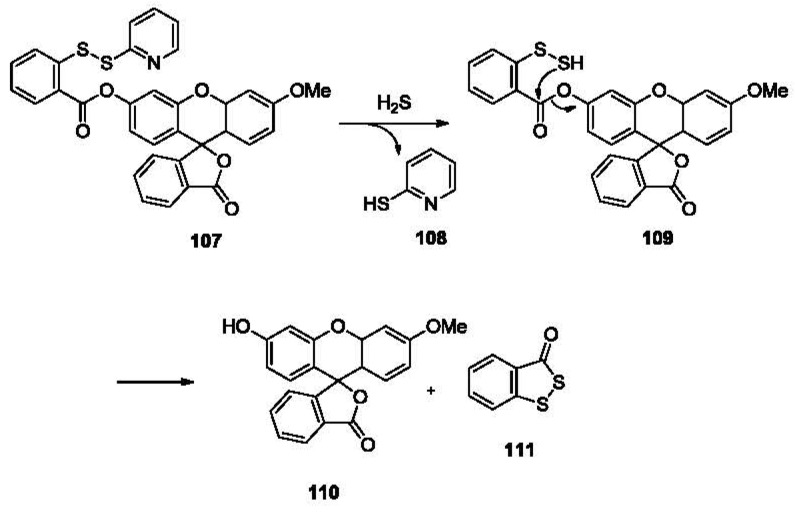
Selective detection of H_2_S based on a disulfide cleavage-cyclization strategy.

Being the simplest molecule in the thiol family, hydrogen sulfide can undergo two deprotonation steps. In other words, it can undergo two nucleophilic reactions. This distinguishes it from other thiols and provides a strategy for sulfide detection with high selectivity. The Xian group reported a fluorescent probe for H_2_S, which was designed based on this principle [164]. As shown in Figure 23, compound **107** reacts with sulfide, releasing mercaptopyridine **108** to form an intermediate **109**, which undergoes a cyclization *in situ* and releases fluorophore **110** and benzodithiolone **111**. This probe is very selective for H_2_S in aqueous solution (PBS/acetonitrile 9:1) among thiols such as Cys and GSH, and gives a linear correlation to sulfide concentrations with a detection limit of low micromolar concentrations. Fluorescent imaging using this probe and exogenous H_2_S has been studied in COS7 cells.

**Figure 24.**
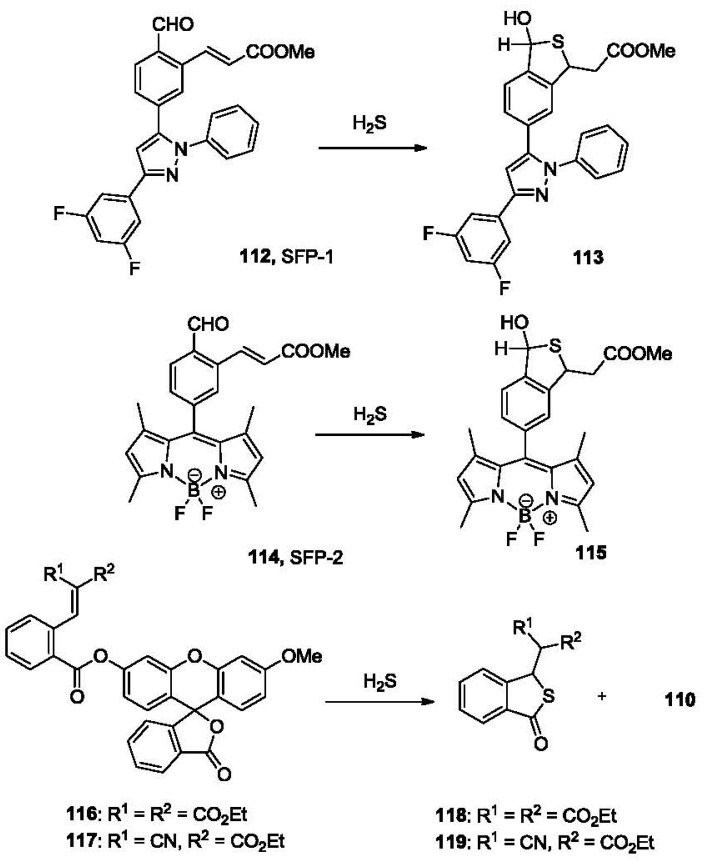
Detection of H_2_S based on Michael addition-cyclization.

Another strategy reported by the He group uses a Michael addition reaction followed by cyclization [165]. In this study, two fluorescent probes, SFP-1 (**112**) and SFP-2 (**114**, Figure 24) were synthesized. These probes bear an α, β-unsaturated ester group at the *ortho* position of a benzaldehyde, which is linked to a fluorophore. The nucleophilic attack by sulfide on the formyl group yields hemithioacetals, which positions the sulfhydryl group for the following Michael addition to form the trapped thioacetal **113** and **115**, in which the PET effect is interrupted and the fluorescence is recovered.

Both SFP-1 and SFP-2 show 50–100 fold selectivity for sulfide over other thiols including β-mercaptoethanol, Cys and GSH. The detection limit is about 5–10 μM with a S/N ratio of 3:1. SFP-2 was used in the *in vivo* imaging of endogenously generated H_2_S triggered by the addition of GSH and Cys in Hela cells. Along a similar line, probes **116** and **117** have been reported recently by the Xian group. These probes are based on a Michael addition-cyclization reaction [166]. In probes **116** and **117**, the Michael acceptor is activated by two electron withdrawing groups. After incubating the probes (5 μM) with 100 μM sulfide for 30 min in phosphate buffer, Michael addition-cyclization takes place to release the fluorophore **110** to form thiolactones **118** and **119**, leading to 11 (**116**) or 160 (**117**)-fold fluorescence increase (λ_ex_ = 465 nm, λ_em_ = 510 nm), respectively. Imaging of exogenous H_2_S was performed in COS7 cells.

Furthermore, chemoprobes developed for other thiols could also be used for H_2_S detection. For example, compound **14** (Figure 3), reported by Maeda for the fluorescent detection of thiols, has also been used for the fluorescent detection of H_2_S [167]. Of course, in such a case, selectivity is an issue.

### 4.2. Probes for H_2_S Based on Reduction Reactions

**Figure 25.**
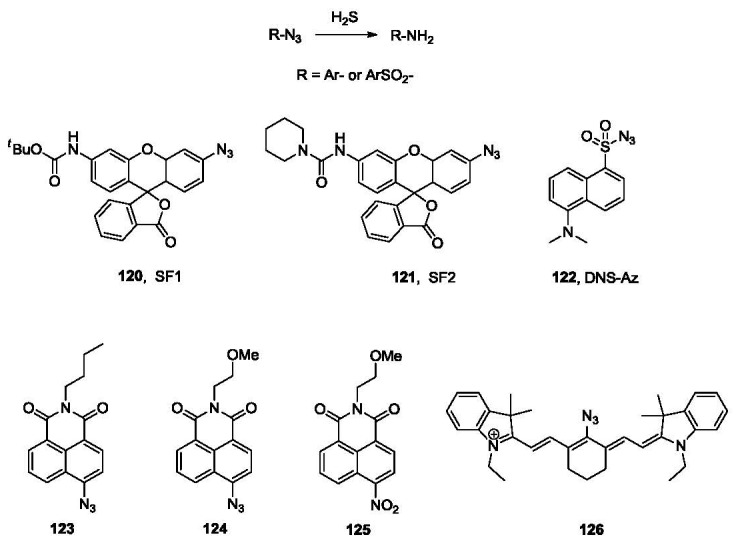
Fluorescent probes for H_2_S based on reduction reactions.

Sulfide is a fairly strong reducing agent. This is another chemical property of H_2_S that can be used in probe design. Azides are known to be reduced by sulfide anion [168]. However, it is not until very recently that this reaction was utilized in the selective detection of sulfide independently by the labs of Chang and Wang. Specifically, the Chang group reported the synthesis and evaluation of two fluorescent probes (SF1, **120** and SF2, **121**, Figure 25) based on this strategy [169]. Both probes bear a fluorescein moiety attached directly to an azido group, which is easily reduced to an amino group by hydrogen sulfide, resulting in a significant increase in fluorescence. The selectivity of these two probes was demonstrated among various RSS (reactive sulfur species), RNS (reactive nitrogen species) and ROS (reactive oxygen species) including GSH, Cys, Na_2_SO_3_, NO, H_2_O_2_ and O^2−^. The probes were also evaluated for *in vivo* cell imaging in HEK293T cells using exogenous H_2_S [169].

At the same time, another fluorescent probe for H_2_S was reported by the Wang group [170]. It is based on the reduction of the azido group attached to a sulfonylnaphthalene fluorophore, the dansyl dye (DNS-Az, **122**, Figure 25). This probe showed a dramatic fluorescence increase (λ_ex_ 340 nm, λ_em_ 517 nm, over 40 fold) upon addition of 25 μM of sulfide into phosphate buffer with Tween-20. The probe was evaluated in different aqueous media, including phosphate buffer and commercial bovine serum. Good selectivity of DNS-Az for H_2_S was observed among various anions (>50 fold) and other reducing agents, including Cys (60-fold) and thiophenol (4-7.5 fold). Another important advantage of this probe over other reported sulfide probes is the fast response [171]. The reduction reaction is complete in seconds in bovine serum at room temperature. This is among the fastest H_2_S fluorescent probes reported so far. Considering the volatility and low stability of H_2_S, an “instant” probe is very important because it allows for accurate detection of sulfide concentration without sample pre-treatment such as precipitation. The fluorescence intensity could be recorded on a fluorometer or a multi-label counter. Excellent linear calibration curves were obtained for H_2_S in different solvents, including buffer and bovine serum. The probe was used for the measurement of H_2_S concentration in blood using the C57BL6/J mouse model. Specifically, a stock solution of DNS-Az was added to the blood, followed by mixing and reaction at room temperature for 5 min. Blood plasma was then obtained by centrifugation and fluorescence intensity was analyzed in a 96-well plate and multi-label counter. The zero point was obtained by trapping sulfide with ZnCl_2_ and the calibration curve was obtained by using an internal standard method. The blood H_2_S concentration obtained (31.9 ± 9.4 μM) is very similar to most previously reported numbers.

Besides DNS-Az, compound **123** has also been synthesized by the Wang group (unpublished results). This probe is composed of an azido group and the naphthalimide fluorophore moiety. The synthesis of this compound consists of two steps from 4-bromo-1,8-naphthalic anhydride through amide formation with *n*-butylamine and substitution of the bromo group by azido using NaN_3_. This compound is sensitive toward sulfide with a 100-fold fluorescence increase (λ_ex_ 420 nm, λ_em_ 530 nm) in response to the addition of 100 μM of H_2_S in a mixed solvent of 1:1 phosphate buffer/acetonitrile. The reaction is complete in 30 s in acetonitrile and 20 min in phosphate buffer/acetonitrile at room temperature. However, the lower solubility and stability of compound **123** compared with **122** may limit its application. Further structure optimization is ongoing to obtain improved fluorescent probes for H_2_S. Two other naphthalimide probes (compounds **124** and **125**, Figure 25) have also been reported recently [172]. Probe **124** is based on the reduction of an azido group and probe **125** is based on the reduction of a nitro group by sulfide. Both probes can be converted to the corresponding 4-aminonaphthalimide (λ_ex_ 432 nm, λ_em_ 542 nm) by reduction. They were found to show more than 10-fold selectivity toward H_2_S over other reactive oxygen, nitrogen, and sulfur species. In PIPES buffer, the reduction reaction is complete in 90 min (**124**) and 45 min (**125**), respectively, upon treatment with 0.5 mM of sulfide. Fluorescent imaging of exogenous H_2_S using these two probes were demonstrated in Hela cells.

Recently, a NIR fluorescent probe (**126**, Figure 25) for H_2_S was reported by Han and co-workers [173]. When the azido group is reduced by H_2_S, a 50-nm red shift from 610 nm to 660 nm in absorption and a 40 nm red shift from 710 nm to 750 nm (λ_ex_ 625 nm) in emission were observed in HEPES buffer. The reaction took 20 min to complete with a micromolar detection limit. This probe was tested in RAW 264.7 macrophage cells for imaging exogenously added H_2_S.

Due to its high selectivity and reactivity, redox-based strategy is becoming very useful in the detection of H_2_S. The level of activities is very high in this area. Recently, several other probes have been reported based on this strategy [174–177]. These probes include two-photon fluorescent probes and protein-based probes.

### 4.3. Probes for H_2_S Based on Metal Sulfide Formation

**Figure 26.**
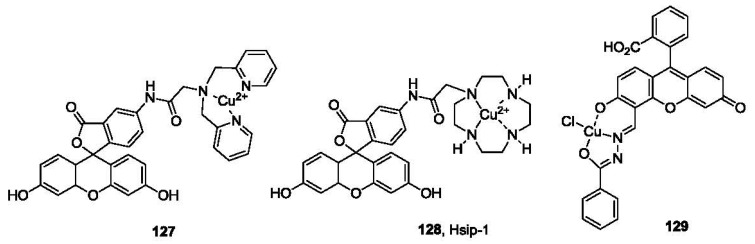
Reagents for the detection of H_2_S based on metal sulfide formation.

The selective interaction between sulfide and copper has been utilized in the detection of sulfide. A fluorescein derivative (**127**, Figure 26) with a dipicolylamine (DPA) binding site for Cu^2+^ was developed by the Chang group [178]. The fluorescence intensity of the DPA conjugate (λ_ex_ 470 nm, λ_em_ 517 nm) was almost completely quenched by Cu^2+^. However, when exposed to sulfide in 10 mM HEPES buffer at pH 7.0, Cu^2+^ was extracted, leading to the formation of CuS and the free fluorophore ligand, and subsequently a significant fluorescence increase. Selectivity for sulfide was demonstrated among various anions. A similar strategy was recently reported by Nagano and co-workers in the development of a fluorescent probe for hydrogen sulfide (Hsip-1, **128**, Figure 26) [179]. Instead of the DPA ligand, cyclen (1,4,7,10-tetraazacyclododecane) was used as the ligand for copper complexation. Hsip-1 exhibited significant fluorescence enhancement (λ_ex_ 491 nm, λ_em_ 516 nm) when exposed to sulfide in HEPES buffer at pH 7.4. Compared with the DPA ligand probe **127**, Hsip-1 showed about 10-fold selectivity for sulfide over GSH. Visualization of H_2_S using Hsip1 was demonstrated in live Hela cells. Recently, another fluorescein based probe (**129**, Figure 26) based on a similar strategy was described [180]. The fluorescence is almost completely quenched by copper. When exposed to sulfide in HEPES/CH_3_CN (pH 7.0, 6: 4, v/v), fluorescence is restored (λ_ex_ 494 nm, λ_em_ 523 nm). Although the selectivity was only 1.6 fold over Cys, the probe was reported to be reversible (up to 10 cycles) upon addition of Cu(II)/H_2_S. This could be used as an on-off-on probe, which could have potential applications for continuous monitoring. Recently, additional probes have been developed based on metal sulfide formation [181–183].

**Figure 27.**
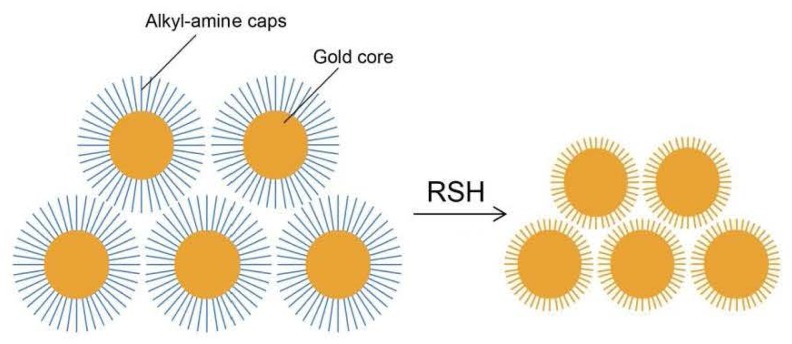
Gaseous mercaptan detection by amine capped Au nanocrystal films.

The selective interaction of sulfur with gold was also utilized in the detection of H_2_S and other thiols. Lewis and co-workers have developed a mercaptan gas detector using amine capped Au nanocrystal films (Figure 27) [184]. The film consists of dodecylamine-capped, 6-7 nm diameter Au nanocrystals. When exposed to gaseous thiols such as CH_3_SH, the amine cap is displaced by thiols, resulting in an electrical resistance drop due to the reduced distance amongst Au cores. The slope of the resistance curve shows a linear response to CH_3_SH between 0.153 and 1.53 ppm in air with the detection limit of 1.7 ppb. Although the film detector does not show a comparable response to larger molecules such as octanethiol, it still provides a very sensitive detection of small molecular gaseous mercaptans such as CH_3_SH and H_2_S.

Because of the biological significance of H_2_S, the value for the quantitative detection of this small molecule has been recognized both for diagnostic applications and in basic research. Fluorescent probes for H_2_S based on nucleophilic cyclization, reduction and metal sulfide formation have been reported. Quantitation of H_2_S was achieved in mouse blood samples with DNS-Az (**122**). Fluorescence imaging of endogenous and exogenous H_2_S was demonstrated by various groups using different cell lines. AuNPs was also used in the detection of mercaptan gas including H_2_S and CH_3_SH. These probes provide new chemical tools for biomedical researchers in their investigation of the functions and properties of H_2_S in biological systems.

## 5. Conclusions

With the widely recognized importance of biological thiols, there has been increasing interest in developing new detection methods. Numerous fluorescent probes have been reported so far for the selective detection of thiols. The probes for biological amino-thiols include those based on nucleophilic substitution, Michael addition, and disulfide/Se-N bond cleavage. Among these probes, nucleophilic substitution-based probes (such as mBrB) are commonly used in the fluorometric/colorimetric labeling of thiols for HPLC quantitation. Disulfide cleavage-based probes (such as Ellman's reagent) are frequently used for estimation of total sulfhydryl groups in a protein sample. Methods based on other probes are still in development. Fluorescent probes for H_2_S include those based on nucleophilic cyclization, reduction, and metal sulfide formation. These probes are emerging as important detection tools of H_2_S. Generally speaking, excellent methods are available for the quantitative and selective concentration determinations of various thiols in solution or in sera. Still needed are methods that are fast enough to address the issues of rapid fluctuations of intracellular concentrations or *in vivo*, especially for hydrogen sulfide. Recently, there is growing interest in the intracellular imaging of various thiols. Fluorescent probes are needed that are: (1) selective, (2) fast response, (3) cell permeable, and (4) of long wavelength. Among these, especially important is the availability of probes that are fast in response because reaction times on the scale of minutes run the risk of perturbing the thiol homeostasis mechanisms in such a way that the amount of probe consumed (and thus fluorescent intensity) may not be correlated with the true concentration at a given time. However, based on the level of research interest in this area, there are good reasons to be optimistic that excellent solutions will be found in the not too distant future.

## Figures and Tables

**Table 1.** A comparison of fluorogenic and chromogenic labeling agents.

**Compound**	**λ_ex_/λ_em_ (nm) or Abs. (nm)**	**Reaction Time (min)**	**Reaction Temperature/pH**	**LOD**	**Biological Sample**	**Refs.**
mBrB	380/480	2–20	25 °C/7.4–8.9	0.5 pM	plasma	[57–60]
ABD-F	380/510	5–60	37–60 °C /7.5–9.3	0.3 pM	plasma, cell	[61–63]
SBD-F	380/510	60	60 °C/9.5–10.5	0.1–1.5 μM	plasma	[64,65]
5-IAF	494/521	15–120	25–40 °C/12.5	2–30 nM	plasma	[66–68]
CMPI	310	15–30	25 °C/8.2	0.3–20 nM	urine	[69,70]
CMQT	355	1–4	25 °C/7.5	50 nM	urine, plasma	[71–73]

## Abbreviations

ABD-F: 4-Fluoro-7-sulfamoylbenzofurazan
ADNB: 5-(2-aminoethyl)dithio-2-nitrobenzoate
AIE: aggregation-induced emission
ATP: adenosine triphosphate
AuNPs: gold nanoparticles
BHMT: betaine-homocysteine S-methyltransferase
BIPM: N-[4-(2-benzimidazolyl)phenyl]maleimide
BODIPY: 4,4-difluoro-4-bora-3a,4a-diaza-s-indacene
CAT: cysteine aminotransferase
CBS: cystathionine β-synthase
CE: capillary electrophoresis
ChE: cholinesterase
CMPI: 2-chloro-1-methylpyridinium iodide
CMQT: 2-chloro-1-methylquinolinium tetrafluoroborate
CNS: central nervous system
CSE: cystathionine γ-lyase or CGL
CV: cardiovascular
Cys: cysteine
DAD: diode array detector
DNBS: 2,4-dinitrophenyl sulfonyl group
D-PEN: D-penicillamine or PenA
DSP: dithiobis(succinimidylpropinate)
DTT: dithiothreitol
DTNB: 5,5′-dithiobis(2-nitrobenzoic acid or Ellman's reagent
EPA: US Environmental Protection Agency
FRET: Förster resonance energy transfer
GCL: γ-glutamate-cysteine ligase or γ-glutamylcysteine synthetase (γ-GCS)
Gly: glycine
Glu: glutamate
GPx: glutathione peroxidase
GSH: glutathione or γ-L-glutamyl-L-cysteinylglycine
GSR: glutathione reductase
GSSG: glutathione disulfide
Hcy: homocysteine
HEPES: 4-(2-hydroxyethyl)-1-piperazineethanesulfonic acid
HPLC: high performance liquid chromatography
5-IAF: 5-iodoacetamidofluorescein
ICT: intramolecular charge transfer
LSPR: localized surface plasmon resonance
LuxS: *S*-ribosylhomocysteinase
MAT: methionine adenosyltransferase
MLCT: metal-to-ligand charge transfer
mBrB: monobromobimane
MES: 2-(*N*-morpholino)ethanesulfonic acid
3MST: 3-mercaptopyruvate sulfurtransferase
MTs: AdoMet-dependent methyltransfersess
*N*-DBPM: *N*-[4-(dimethylamino-2-benzofuranyl)phenyl]maleimide
NIR: near infrared
ODNB: 5-octyldithio-2-nitrobenzoate
PBS: phosphate buffered saline
2-PDS: 2,2′-dipyridyl disulfide
PET: photoinduced electron transfer
PIPES: piperazine-*N,N*′-bis(2-ethanesulfonic acid)
RNS: reactive nitrogen species
ROS: reactive oxygen species
RSS: reactive sulfur species
SAHH: S-adenosylhomocysteine hydrolase
SBD-F: 7-Fluorobenzo-2-oxa-1,3-diazole-4-sulfonic acid ammonium salt
Ser: serine
TCDI: 1,1′-thiocarbonyldiimidazole
TCEP: tris(2-carboxyethyl)phosphine
tHcy: total Hcy concentration
TNB: 5-thio-2-nitrobenzoate
TP: tiopronin or Thiola
2-TP: 2-thiopyridone
4-TP: 4-thiopyridone
TPM: two-photon microscopy
TPP: triphenylphosphonium
Trx: thioredoxin
UV: ultraviolet

## References

[1] Koval I.V. (2007). Reactions of thiols. Russ. J. Org. Chem..

[2] Meister A., Anderson M.E. (1983). Glutathione. Ann. Rev. Biochem..

[3] Selhub J. (1999). Homocysteine metabolism. Annu. Rev. Nutr..

[4] Forman H.J., Zhang H.Q., Rinna A. (2009). Glutathione: Overview of its protective roles, measurement, and biosynthesis. Mol. Aspects Med..

[5] Cleland W.W. (1964). Dithiothreitol, a new protective reagent for SH groups. Biochemistry.

[6] Lipton S.A., Choi Y.B., Takahashi H., Zhang D.X., Li W.Z., Godzik A., Bankston L.A. (2002). Cysteine regulation of protein function—As exemplified by NMDA-receptor modulation. Trends Neurosci..

[7] Petsko G.A., Ringe D. (2004). Protein Structure and Function: From Sequence to Consequence.

[8] Meister A. (1988). Glutathione metabolism and its selective modification. J. Biol. Chem..

[9] Conklin K.A. (2004). Chemotherapy-associated oxidative stress: impact on chemotherapeutic effectiveness. Integr. Cancer. Ther..

[10] Peisach J., Blumberg W.E. (1969). A mechanism for the action of penicillamine in the treatment of Wilson's disease. Mol. Pharmacol..

[11] Zhang J.G., Lindup W.E. (1996). Tiopronin protects against the nephrotoxicity of cisplatin in rat renal cortical slices *in vitro*. Toxicol. Appl. Pharm..

[12] Boehning D., Snyder S.H. (2003). Novel neural modulators. Annu. Rev. Neurosci..

[13] Martelli A., Testai L., Breschi M.C., Blandizzi C., Virdis A., Teddei S., Calderone V. (2011). Hydrogen sulfide: novel oppotunity for drug discovery. Med. Res. Rev..

[14] Szabo C. (2007). Hydrogen sulphide and its therapeutic potential. Nat. Rev. Drug Discov..

[15] Wang R. (2002). Two's company, three's a crowd: Can H_2_S be the third endogenous gaseous transmitter?. FASEB J..

[16] Kluijtmans L.A.J., van den Heuvel L.P.W.J., Boers G.H.J., Frosst P., Stevens E.M.B., vanOost B.A., den Heijer M., Trijbels F.J.M., Rozen R., Blom H.J. (1996). Molecular genetic analysis in mild hyperhomocysteinemia: A common mutation in the methylenetetrahydrofolate reductase gene is a genetic risk factor for cardiovascular disease. Am. J. Hum. Genet..

[17] Calonge M.T., Gasparini P., Chillaron J., Chillon M., Gallucci M., Rousaud F., Zelante L., Testar X., Dallapiccola B., Disilverio F. (1994). Cystinuria caused by mutations in rbat, a gene involved in the transport of cystine. Nat. Genet..

[18] Lowicka E., Beltowski J. (2007). Hydrogen sulfide (H_2_S)—The third gas of interest for pharmacologists. Pharmacol. Rep..

[19] Wu G.Y., Fang Y.Z., Yang S., Lupton J.R., Turner N.D. (2004). Glutathione metabolism and its implications for health. J. Nutr..

[20] Droge W., Eck H.P., Mihm S. (1992). Hiv-Induced Cysteine Deficiency and T-Cell Dysfunction—A rationale for treatment with N-Acetylcysteine. Immunol. Today.

[21] Refsum H., Smith A.D., Ueland P.M., Nexo E., Clarke R., McPartlin J., Johnston C., Engbaek F., Schneede J., McPartlin C. (2004). Facts and recommendations about total homocysteine determinations: An expert opinion. Clin. Chem..

[22] Levy H.L., Kraus J.P., Mudd S.H., Valle D., Beaudet A.L., Vogelstein B., Kinzler K.W., Antonarakis S.E., Ballabio A., Scriver C.R., William S.S., Childs B. (1995). Disorders of Transsulfuration. The Metabolic and Molecular Bases of Inherited Disease.

[23] Chadefaux B., Ceballos I., Hamet M., Coude M., Poissonnier M., Kamoun P., Allard D. (1988). Is absence of atheroma in Down syndrome due to decreased homocysteine levels?. Lancet.

[24] James S.J., Pogribna M., Melnyk S., Pogribny I., Chango A., Yi P. (2001). Homocysteine metabolism in children with Down syndrome: *In vitro* modulation. Am. J. Hum. Genet..

[25] Kang S.S., Wong P.W.K., Norusis M. (1987). Homocysteinemia due to folate deficiency. Metabolism.

[26] Klee G.G. (2000). Cobalamin and folate evaluation: Measurement of methylmalonic acid and homocysteine vs vitamin B-12 and folate. Clin. Chem..

[27] Ubbink J.B., van der Merwe A., Delport R., Allen R.H., Stabler S.P., Riezler R., Vermaak W.J.H. (1996). The effect of a subnormal vitamin B-6 status on homocysteine metabolism. J. Clin. Invest..

[28] Medina M.A., Amores-Sanchez M.I. (2000). Homocysteine: an emergent cardiovascular risk factor?. Eur. J. Clin. Invest..

[29] Bostom A.G., Culleton B.F. (1999). Hyperhomocysteinemia in chronic renal disease. J. Am. Soc. Nephrol..

[30] Jones D.P., Carlson J.L., Samiec P.S., Sternberg P., Mody V.C., Reed R.L., Brown L.A.S. (1998). Glutathione measurement in human plasma Evaluation of sample collection, storage and derivatization conditions for analysis of dansyl derivatives by HPLC. Clin. Chim. Acta.

[31] Akerboom T.P.M., Bilzer M., Sies H. (1982). Relationship of biliary glutathione disulfide efflux and intra cellular glutathione disulfide content in perfused rat liver. J. Biol. Chem..

[32] Griffith O.W. (1999). Biologic and pharmacologic regulation of mammalian glutathione synthesis. Free Radic. Bio. Med..

[33] Rahman I., Biswas S.K. (2009). Environmental toxicity, redox signaling and lung inflammation: The role of glutathione. Mol. Aspects Med..

[34] Kruger W.D., Chen X.L., Jhee K.H. (2004). Production of the neuromodulator H_2_S by cystathionine beta-synthase via the condensation of cysteine and homocysteine. J. Biol. Chem..

[35] Ishii I., Akahoshi N., Yu X.N., Kobayashi Y., Namekata K., Komaki G., Kimura H. (2004). Murine cystathionine gamma-lyase: Complete cDNA and genomic sequences, promoter activity, tissue distribution and developmental expression. Biochem. J..

[36] Shibuya N., Mikami Y., Kimura Y., Nagahara N., Kimura H. (2009). Vascular endothelium expresses 3-mercaptopyruvate sulfurtransferase and produces hydrogen sulfide. J. Biochem..

[37] Tanizawa K. (2011). Production of H_2_S by 3-mercaptopyruvate sulphurtransferase. J. Biochem..

[38] Calvert J.W., Jha S., Gundewar S., Elrod J.W., Ramachandran A., Pattillo C.B., Kevil C.G., Lefer D.J. (2009). Hydrogen sulfide mediates cardioprotection through Nrf2 signaling. Circ. Res..

[39] Calvert J.W., Elston M., Nicholson C.K., Gundewar S., Jha S., Elrod J.W., Ramachandran A., Lefer D.J. (2010). Genetic and pharmacologic hydrogen sulfide therapy attenuates ischemia-induced heart failure in mice. Circulation.

[40] Zhao W.M., Zhang J., Lu Y.J., Wang R. (2001). The vasorelaxant effect of H_2_S as a novel endogenous gaseous K-ATP channel opener. EMBO J..

[41] Lefer D.J. (2007). A new gaseous signaling molecule emerges: Cardioprotective role of hydrogen sulfide. P. Natl. Acad. Sci. USA.

[42] Abe K., Kimura H. (1996). The possible role of hydrogen sulfide as an endogenous neuromodulator. J. Neurosci..

[43] Kimura H. (2002). Hydrogen sulfide as a neuromodulator. Mol. Neurobiol..

[44] Moore P.K., Bhatia M., Moochhala S. (2003). Hydrogen sulfide: from the smell of the past to the mediator of the future?. Trends Pharmacol. Sci..

[45] Elrod J.W., Calvert J.W., Morrison J., Doeller J.E., Kraus D.W., Tao L., Jiao X.Y., Scalia R., Kiss L., Szabo C. (2007). Hydrogen sulfide attenuates myocardial ischemia-reperfusion injury by preservation of mitochondrial function. Proc. Natl. Acad. Sci. USA.

[46] Sen U., Mishra P.K., Tyagi N., Tyagi S.C. (2010). Homocysteine to hydrogen sulfide or hypertension. Cell Biochem. Biophys..

[47] Kamoun P., Belardinelli M.-C., Chabli A., Lallouchi K. (2003). Chadefaux-Vekemans, B. Endogenous hydrogen sulfide overproduction in down syndrom. Am. J. Med. Genet..

[48] Chen Y.-H., Yao W.-Z., Geng B., Ding Y.-L., Lu M., Zhao M.-W., Tang C.-S. (2005). Endogenous hydrogen sulfide in patients with COPD. Chest.

[49] Aitken S.M., Kirsch J.F. (2003). Kinetics of the yeast cystathionine beta-synthase forward and reverse reactions: Continuous assays and the equilibrium constant for the reaction. Biochemistry.

[50] Zhu J.G., Dizin E., Hu X.B., Wavreille A.S., Park J., Pei D.H. (2003). S-ribosylhomocysteinase (LuxS) is a mononuclear iron protein. Biochemistry.

[51] Ellman G.L., Courtney K.D., Andres V., Feather-Stone R.M. (1961). A new and rapid colorimetric determination of acetylcholinesterase activity. Biochem. Pharmacol..

[52] Brown K.S., Kingsbury W.D., Hall N.M., Dunn G.L., Gilvarg C. (1987). Determination of carboxypeptidase A using N-acetyl-phenylalanyl-3-thiaphenylalanine as substrate application to a direct serum assay. Anal. Biochem..

[53] Chen X., Zhou Y., Peng X.J., Yoon J. (2010). Fluorescent and colorimetric probes for detection of thiols. Chem. Soc. Rev..

[54] Yin L.L., Chen Z.Z., Tong L.L., Xu K.H., Tang B. (2009). Progress on fluorescent probes for thiols. Chinese J. Anal. Chem..

[55] Toyo'oka T. (2009). Review Recent advances in separation and detection methods for thiol compounds in biological samples. J. Chromatogr. B..

[56] Nekrassova O., Lawrence N.S., Compton R.G. (2003). Analytical determination of homocysteine: A review. Talanta.

[57] Sakhi A.K., Blomhoff R., Gundersen T.E. (2007). Simultaneous and trace determination of reduced and oxidized glutathione in minute plasma samples using dual mode fluorescence detection and column switching high performance liquid chromatography. J. Chromatogr. A.

[58] Ivanov A.R., Nazimov I.V., Baratova L.A. (2000). Qualitative and quantitative determination of biologically active low-molecular-mass thiols in human blood by reversed-phase high-performance liquid chromatography with photometry and fluorescence detection. J. Chromatogr. A.

[59] Kamencic H., Lyon A., Paterson P.G., Juurlink B.H.J. (2000). Monochlorobimane fluorometric method to measure tissue glutathione. Anal. Biochem..

[60] Chou S.T., Ko L.E., Yang C.S. (2001). High performance liquid chromatography with fluorimetric detection for the determination of total homocysteine in human plasma: Method and clinical applications. Anal. Chim. Acta.

[61] Toyo'oka T., Tanabe J., Kashihara Y. (2001). Determination of intracellular glutathione in rat hepatocytes after treatment of environmental pollutants by capillary electrophoresis with laser-induced fluorescence detection. Anal. Chim. Acta.

[62] Ogasawara Y., Mukai Y., Togawa T., Suzuki T., Tanabe S., Ishii K. (2007). Determination of plasma thiol bound to albumin using affinity chromatography and high-performance liquid chromatography with fluorescence detection: Ratio of cysteinyl albumin as a possible biomarker of oxidative stress. J. Chromatogr. B..

[63] Satoh S., Shindoh M., Min J.Z., Toyo'oka T., Fukushima T., Inagaki S. (2008). Selective and sensitive determination of lipoyllysine (protein-bound alpha-lipoic acid) in biological specimens by high-performance liquid chromatography with fluorescence detection. Anal. Chim. Acta.

[64] Feussner A., Rolinski B., Weiss N., Deufel T., Wolfram G., Roscher A.A. (1997). Determination of total homocysteine in human plasma by isocratic high-performance liquid chromatography. Eur. J. Clin. Chem. Clin..

[65] Nolin T.D., McMenamin M.E., Himmelfarb J. (2007). Simultaneous determination of total homocysteine, cysteine, cysteinylglycine, and glutathione in human plasma by high-performance liquid chromatography: Application to studies of oxidative stress. J. Chromatogr. B..

[66] Carru C., Deiana L., Sotgia S., Pes G.M., Zinellu A. (2004). Plasma thiols redox status by laser-induced fluorescence capillary electrophoresis. Electrophoresis.

[67] Zinellu A., Carru C., Sotgia S., Deiana L. (2004). Plasma D-penicillamine redox state evaluation by capillary electrophoresis with laser-induced fluorescence. J. Chromatogr. B..

[68] Musenga A., Mandrioli R., Bonifazi P., Kenndler E., Pompei A., Raggi M.A. (2007). Sensitive and selective determination of glutathione in probiotic bacteria by capillary electrophoresis-laser induced fluorescence. Anal. Bioanal. Chem..

[69] Kaniowska E., Chwatko G., Glowacki R., Kubalczyk P., Bald E. (1998). Urinary excretion measurement of cysteine and homocysteine in the form of their S-pyridinium derivatives by high-performance liquid chromatography with ultraviolet detection. J. Chromatogr. A.

[70] Bald E., Kaniowska E., Chwatko G., Glowacki R. (2000). Liquid chromatographic assessment of total and protein-bound homocysteine in human plasma. Talanta.

[71] Kuimierek K., Bald E. (2008). Measurement of reduced and total mercaptamine in urine using liquid chromatography with ultraviolet detection. Biomed. Chromatogr..

[72] Kubalczyk P., Bald E. (2006). Transient pseudo-isotachophoretic stacking in analysis of plasma for homocysteine by capillary zone electrophoresis. Anal. Bioanal. Chem..

[73] Bald E., Chwatko G., Glowacki R., Kusmierek K. (2004). Analysis of plasma thiols by high-performance liquid chromatography with ultraviolet detection. J. Chromatogr. A.

[74] Federici G., Pastore A., Massoud R., Motti C., Lo Russo A., Fucci G., Cortese C. (1998). Fully automated assay for total homocysteine, cysteine, cysteinylglycine, glutathione, cysteamine, and 2-mercaptopropionylglycine in plasma and urine. Clin. Chem..

[75] Imai K., Toyo'oka T. (1987). Fluorometric assay of thiols with fluorobenzoxadiazoles. Methods Enzymol..

[76] Toyo'oka T., Tanabe J., Jinno H. (2001). Determination of rat hepatocellular glutathione by reversed-phase liquid chromatography with fluorescence detection and cytotoxicity evaluation of environmental pollutants based on the concentration change. Biomed. Chromatogr..

[77] Satoh S., Toyo'oka T., Fukushima T., Inagaki S. (2007). Simultaneous determination of alpha-lipoic acid and its reduced form by high-performance liquid chromatography with fluorescence detection. J. Chromatogr. B..

[78] Kusmierek K., Glowacki R., Bald E. (2006). Analysis of urine for cysteine, cysteinylglycine, and homocysteine by high-performance liquid chromatography. Anal. Bioanal. Chem..

[79] Kusmierek K., Bald E. (2007). Simultaneous determination of tiopronin and D-penicillamine in human urine by liquid chromatography with ultraviolet detection. Anal. Chim. Acta.

[80] Huang T.M., Yang B., Yu Y.J., Zheng X.W., Duan G.L. (2006). Reverse-phase high performance liquid chromatography for the determination of tiopronin in human plasma after derivatization with p-bromophenacyl bromide. Anal. Chim. Acta.

[81] Maeda H., Matsuno H., Ushida M., Katayama K., Saeki K., Itoh N. (2005). 2,4-Dinitrobenzenesulfonyl fluoresceins as fluorescent alternatives to Ellman's reagent in thiol-quantification enzyme assays. Angew. Chem. Int. Ed..

[82] Bouffard J., Kim Y., Swager T.M., Weissleder R., Hilderbrand S.A. (2008). A highly selective fluorescent probe for thiol bioimaging. Org. Lett..

[83] Wang S.-P., Deng W.-J., Sun D., Yan M., Zheng H., Xu J.-G. (2009). A colorimetric and fluorescent merocyanine-based probe for biological thiols. Org. Biomol. Chem..

[84] Li X., Qian S., He Q., Yang B., Li J., Hu Y. (2010). Design and synthesis of a highly selective fluorescent turn-on probe for thiol bioimaging in living cells. Org. Biomol. Chem..

[85] Shao J., Guo H., Ji S., Zhao J. (2011). Styryl-BODIPY based red-emitting fluorescent OFF–ON molecular probe for specific detection of cysteine. Biosens. Bioelectron..

[86] Ji S., Guo H., Yuan X., Li X., Ding H., Gao P., Zhao C., Wu W., Wu W., Zhao J. (2010). A highly selective off-on red-emitting phosphorescent thiol probe with large stokes shift and long luminescent lifetime. Org. Lett..

[87] Ercal N., Yang P., Aykin N. (2001). Determination of biological thiols by high-performance liquid chromatography following derivatization by ThioGlo maleimide reagents. J. Chromatogr. B.

[88] Matsumoto T., Urano Y., Shoda T., Kojima H., Nagano T. (2007). A thiol-reactive fluorescence probe based on donor-excited photoinduced electron transfer: Key role of ortho substitution. Org. Lett..

[89] Guo X.-F., Wang H., Guo Y.-H., Zhang H.-S. (2009). Selective spectrofluorimetric determination of glutathione in clinical and biological samples using 1,3,5,7-tetramethyl-8-phenyl-(2-maleimide)-difluoroboradiaza-s-indacene. Anal. Chim. Acta.

[90] Liu Y., Yu Y., Lam J.W.Y., Hong Y., Faisal M., Yuan W.Z., Tang B.Z. (2010). Simple biosensor with high selectivity and sensitivity: Thiol-specific biomolecular probing and intracellular imaging by AIE fluorogen on a TLC plate through a thiol–ene click mechanism. Chem. Eur. J..

[91] Kand D., Kalle A.M., Varma S.J., Talukdar P. (2012). A chromenoquinoline-based fluorescent off-on thiol probe for bioimaging. Chem. Commun..

[92] Zeng Y., Zhang G., Zhang D. (2008). A selective colorimetric chemosensor for thiols based on intramolecular charge transfer mechanism. Anal. Chim. Acta.

[93] Yi L., Li H.Y., Sun L., Liu L.L., Zhang C.H., Xi Z. (2009). A Highly Sensitive Fluorescence Probe for Fast Thiol-Quantification Assay of Glutathione Reductase. Angew. Chem. Int. Ed..

[94] Lin W., Yuan L., Cao Z., Feng Y., Long L. (2009). A sensitive and selective fluorescent thiol probe in water based on the conjugate 1,4-addition of thiols to α,β-unsaturated ketones. Chem. Eur. J..

[95] Kwon H., Lee K., Kim H.-J. (2011). Coumarin-malonitrile conjugate as a fluorescence turn-on probe for biothiols and its cellular expression. Chem. Commun..

[96] Ros-Lis J.V., García B., Jiménez D., Martínez-Máñez R., Sancenón F., Soto J., Gonzalvo F., Valldecabres M.C. (2004). Squaraines as fluoro−chromogenic probes for thiol-containing compounds and their application to the detection of biorelevant thiols. J. Am. Chem. Soc..

[97] Stolik S., Delgado J.A., Perez A., Anasagasti L. (2000). Measurement of the penetration depths of red and near infrared light in human “*ex vivo*” tissues. J. Photochem. Photobiol. B.

[98] Sreejith S., Divya K.P., Ajayaghosh A. (2008). A near-infrared squaraine dye as a latent ratiometric fluorophore for the detection of aminothiol content in blood plasma. Angew. Chem. Int. Ed..

[99] Ros-Lis J.V., Martinez-Manez R., Soto J. (2002). A selective chromogenic reagent for cyanide determination. Chem. Commun..

[100] Huo F.-J., Sun Y.-Q., Su J., Chao J.-B., Zhi H.-J., Yin C.-X. (2009). Colorimetric detection of thiols using a chromene molecule. Org. Lett..

[101] Chen X., Ko S.-K., Kim M.J., Shin I., Yoon J. (2010). A thiol-specific fluorescent probe and its application for bioimaging. Chem. Commun..

[102] Riddles P.W., Blakeley R.L., Zerner B. (1983). Reassessment of Ellman's reagent. Methods Enzymol..

[103] Taylan E., Resmi H. (2010). The Analytical Performance of a Microplate Method for Total Sulfhydryl Measurement in Biological Samples. Turk. J. Biochem..

[104] Ellman G.L. (1959). Tissue sulfhydryl groups. Arch. Biochem. Biophys..

[105] Eyer P., Worek F., Kiderlen D., Sinko G., Stuglin A., Simeon-Rudolf V., Reiner E. (2003). Molar absorption coefficients for the reduced Ellman reagent: reassessment. Anal. Biochem..

[106] Faulstich H., Tews P., Heintz D. (1993). Determination and derivatization of protein thiols by N-octyldithionitrobenzoic acid. Anal. Biochem..

[107] Zhang H., Le M., Means G.E. (1998). A kinetic approach to characterize the electrostatic environments of thiol groups in proteins. Bioorg. Chem..

[108] Zhu J.G., Dhimitruka I., Pei D. (2004). 5-(2-aminoethyl)dithio-2-nitrobenzoate as a more base-stable alternative to Ellman's reagent. Org. Lett..

[109] Le M., Means G.E. (1995). A procedure for the determination of monothiols in the presence of dithiothreitol -an improved assay for the reduction of disulfides. Anal. Biochem..

[110] Grassetti D.R., Murray J.F. (1967). Determination of sulfhydryl groups with 2,2′- or 4,4′-dithiodipyridine. Arch. Biochem. Biophys..

[111] Riener C.K., Kada G., Gruber H.J. (2002). Quick measurement of protein sulfhydryls with Ellman's reagent and with 4,4′-dithiodipyridine. Anal. Bioanal. Chem..

[112] Cao X.W., Lin W.Y., Yu Q.X. (2011). A ratiometric fluorescent probe for thiols based on a tetrakis (4-hydroxyphenyl)porphyrin-coumarin scaffold. J. Org. Chem..

[113] Pires M.M., Chmielewski J. (2008). Fluorescence imaging of cellular glutathione using a latent rhodamine. Org. Lett..

[114] Denk W., Strickler J.H., Webb W.W. (1990). Two-photon laser scanning fluorescence microscopy. Science.

[115] Rubart M. (2004). Two-photon microscopy of cells and tissue. Circ. Res..

[116] Helmchen F., Denk W. (2005). Deep tissue two-photon microscopy. Nat. Methods.

[117] Zipfel W.R., Williams R.M., Webb W.W. (2003). Nonlinear magic: multiphoton microscopy in the biosciences. Nat. Biotechnol..

[118] Lee J.H., Lim C.S., Tian Y.S., Han J.H., Cho B.R. (2010). A two-photon fluorescent probe for thiols in live cells and tissues. J. Am. Chem. Soc..

[119] Lim C.S., Masanta G., Kim H.J., Han J.H., Kim H.M., Cho B.R. (2011). Ratiometric detection of mitochondrial thiols with a two-photon fluorescent probe. J. Am. Chem. Soc..

[120] Yousif L.F., Stewart K.M., Kelley S.O. (2009). Targeting mitochondria with organelle-specific compounds: Strategies and applications. Chembiochem.

[121] Lee M.H., Han J.H., Kwon P.-S., Bhuniya S., Kim J.Y., Sessler J.L., Kang C., Kim J.S. (2012). Hepatocyte-targeting single galactose-appended naphthalimide: A tool for intracellular thiol imaging *in vivo*. J. Am. Chem. Soc..

[122] Mutus B., Durocher S., Rezaee A., Hamm C., Rangan C., Mittler S. (2009). Disulfide-linked, gold nanoparticle based reagent for detecting small molecular weight thiols. J. Am. Chem. Soc..

[123] Rotruck J.T., Pope A.L., Ganther H.E., Swanson A.B., Hafeman D.G., Hoekstra W.G. (1973). Selenium: Biochemical role as a component of glutathione peroxidase. Science.

[124] Bhabak K.P., Mugesh G. (2010). Functional mimics of glutathione peroxidase: Bioinspired synthetic antioxidants. Acc. Chem. Res..

[125] Mugesh G., du Mont W.W., Sies H. (2001). Chemistry of biologically important synthetic organoselenium compounds. Chem. Rev..

[126] Müller A., Cadenas E., Graf P., Sies H. (1984). A novel biologically active seleno-organic compound-I. Glutathione peroxidase-like activity *in vitro* and antioxidant capacity of PZ 51 (Ebselen). Biochem. Pharmacol..

[127] Tang B., Xing Y.L., Li P., Zhang N., Yu F.B., Yang G.W. (2007). A rhodamine-based fluorescent probe containing a Se-N bond for detecting thiols and its application in living cells. J. Am. Chem. Soc..

[128] Tang B., Yin L., Wang X., Chen Z., Tong L., Xu K. (2009). A fast-response, highly sensitive and specific organoselenium fluorescent probe for thiols and its application in bioimaging. Chem. Commun..

[129] Goodman S.I., Elsas L.J., Rosenblatt D.S. (1998). ASHG/ACMG STATEMENT—Measurement and use of total plasma homocysteine. Am. J. Hum. Genet..

[130] Amarnath V., Amarnath K. (2002). Specific determination of cysteine and penicillamine through cyclization to 2-thioxothiazolidine-4-carboxylic acids. Talanta.

[131] Amarnath K., Amarnath V., Valentine H.L., Valentine W.M. (2003). A specific HPLC-UV method for the determination of cysteine and related aminothiols in biological samples. Talanta.

[132] Strongin R.M., Rusin O., St Luce N.N., Agbaria R.A., Escobedo J.O., Jiang S., Warner I.M., Dawan F.B., Lian K. (2004). Visual detection of cysteine and homocysteine. J. Am. Chem. Soc..

[133] Rusin O., Wang W.H., Xu X.Y., Kim K.K., Escobedo J.O., Fakayode S.O., Fletcher K.A., Lowry M., Schowalter C.M., Lawrence C.M. (2005). Detection of homocysteine and cysteine. J. Am. Chem. Soc..

[134] Tanaka F., Mase N., Barbas C.F. (2004). Determination of cysteine concentration by fluorescence increase: Reaction of cysteine with a fluorogenic aldehyde. Chem. Commun..

[135] Lee K.S., Kim T.K., Lee J.H., Kim H.J., Hong J.I. (2008). Fluorescence turn-on probe for homocysteine and cysteine in water. Chem. Commun..

[136] Yang Z., Zhao N., Sun Y., Miao F., Liu Y., Liu X., Zhang Y., Ai W., Song G., Shen X., Yu X., Sun J., Wong W.-Y. (2012). Highly selective red- and green-emitting two-photon fluorescent probes for cysteine detection and their bio-imaging in living cells. Chem. Commun..

[137] Chen H., Zhao Q., Wu Y., Li F., Yang H., Yi T., Huang C. (2007). Selective phosphorescence chemosensor for homocysteine based on an iridium(III) complex. Inorg. Chem..

[138] Strongin R.M., Wang W.H., Escobedo J.O., Lawrence C.M. (2004). Direct detection of homocysteine. J. Am. Chem. Soc..

[139] Escobedo J.O., Wang W.H., Strongin R.M. (2006). Use of a commercially available reagent for the selective detection of homocysteine in plasma. Nat. Protoc..

[140] Yang X., Guo Y., Strongin R.M. (2011). Conjugate addition/cyclization sequence enables selective and simultaneous fluorescence detection of cysteine and homocysteine. Angew. Chem. Int. Ed..

[141] Guo Z., Nam S., Park S., Yoon J. (2012). A highly selective ratiometric near-infrared fluorescent cyanine sensor for cysteine with remarkable shift and its application in bioimaging. Chem. Sci..

[142] Harris R.L., Bingham E., Cohrssen B. (2001). Patty's Industrial Hygiene and Toxicology.

[143] Hathaway G.J., Proctor N.H. (2004). Proctor and Hughes' Chemical Hazards of the Workplase.

[144] Wang W., Jiang W., Fu Q.Q., Fan H.Y., Ho J. (2007). A highly selective fluorescent probe for thiophenols. Angew. Chem. Int. Ed..

[145] Wang W., Jiang W., Cao Y.T., Liu Y.A. (2010). Rational design of a highly selective and sensitive fluorescent PET probe for discrimination of thiophenols and aliphatic thiols. Chem. Commun..

[146] Lin W.Y., Long L.L., Tan W. (2010). A highly sensitive fluorescent probe for detection of benzenethiols in environmental samples and living cells. Chem. Commun..

[147] Lawrence N.S., Davis J., Compton R.G. (2000). Analytical strategies for the detection of sulfide: A review. Talanta.

[148] Hughes M.N., Centelles M.N., Moore K.P. (2009). Making and working with hydrogen sulfide the chemistry and generation of hydrogen sulfide *in vitro* and its measurement *in vivo*: A review. Free Radic. Biol. Med..

[149] Radfordknoery J., Cutter G.A. (1993). Determination of carbonyl sulfide and hydrogen sulfide species in natural waters using specialized collection procedures and gas chromatography with flame photometric detection. Anal. Chem..

[150] Berube P.R., Parkinson P.D., Hall E.R. (1999). Measurement of reduced sulphur compounds contained in aqueous matrices by direct injection into a gas chromatograph with a flame photometric detector. J. Chromatogr. A.

[151] Hill P.G., Smith R.M. (2000). Determination of sulphur compounds in beer using headspace solid-phase microextraction and gas chromatographic analysis with pulsed flame photometric detection. J. Chromatogr. A.

[152] Hyspler R., Tichá A., Indrová M., Zadák Z., Hysplerová L., Gasparic J., Churácek J. (2002). A simple, optimized method for the determination of sulphide in whole blood by GC–MS as a marker of bowel fermentation processes. J. Chromatogr. B..

[153] Savage J.C., Gould D.H. (1990). Determination of sulfide in brain tissue and rumen fluid by ion-interaction reversed-phase high-performance liquid chromatography. J. Chromatogr. Biomed..

[154] Schiavon G., Zotti G., Toniolo R., Bontempelli G. (1995). Electrochemical detection of trace hydrogen-sulfide in gaseous samples by porous silver electrodes supported on ion-exchange membranes (solid polymer electrolytes). Anal. Chem..

[155] Spilker B., Randhahn J., Grabow H., Beikirch H., Jeroschewski P. (2008). New electrochemical sensor for the detection of hydrogen sulfide and other redox active species. J. Electroanal. Chem..

[156] Doeller J.E., Isbell T.S., Benavides G., Koenitzer J., Patel H., Patel R.P., Lancaster J.R., Darley-Usmar V.M., Kraus D.W. (2005). Polarographic measurement of hydrogen sulfide production and consumption by mammalian tissues. Anal. Biochem..

[157] Li L., Bhatia M., Zhu Y.Z., Ramnath R.D., Wang Z.J., Anuar F.B. M., Whiteman M., Salto-Tellez M., Moore P.K. (2005). Hydrogen sulfide is a novel mediator of lipopolysaccharide-induced inflammation in the mouse. FASEB J..

[158] Kamoun P. (2004). Endogenous production of hydrogen sulfide in mammals. Amino Acids.

[159] Whitfield N.L., Kreimier E.L., Verdial F.C., Skovgaard N., Olson K.R. (2008). Reappraisal of H_2_S/sulfide concentration in vertebrate blood and its potential significance in ischemic preconditioning and vascular signaling. Am. J. Physiol. Regul. Integr. Comp. Physiol..

[160] Lei W., Dasgupta P.K. (1989). Determination of sulfide and mercaptans in caustic scrubbing liquor. Anal. Chim. Acta.

[161] Fonselius S., Dyrssen D., Yhlen B., Grasshoff K., Kremling K., Ehrhardt M. (2007). Determination of Hydrogen Sulphide. Methods of Seawater Analysis.

[162] Sowmya S., Swathi Y., Yeo A.L., Shoon M.L., Moore P.K., Bhatia M. (2010). Hydrogen sulfide: regulatory role on blood pressure in hyperhomocysteinemia. Vasc. Pharmacol..

[163] Lewis G.N., Godschmid O., Magel T.T., Bigeleisen J. (1943). Dimetric and other forms of methylene blue: absorption and fluorescence of the pure monomer. J. Am. Chem. Soc..

[164] Liu C., Pan J., Li S., Zhao Y., Wu L.Y., Berkman C.E., Whorton A.R., Xian M. (2011). Capture and visualization of hydrogen sulfide by a fluorescent probe. Angew. Chem. Int. Ed..

[165] Qian Y., Karpus J., Kabil O., Zhu H.-L., Banerjee R., Zhao J., He C. (2011). Selective fluorescent probes for live-cell monitoring of sulfide. Nat. Commun..

[166] Liu C., Peng B., Li S., Park C.-M., Whorton A.R., Xian M. (2012). Reaction based fluorescent probes for hydrogen sulfide. Org. Lett..

[167] Yang X.F., Wang L.P., Xu H.M., Zhao M.L. (2009). A fluorescein-based fluorogenic and chromogenic chemodosimeter for the sensitive detection of sulfide anion in aqueous solution. Anal. Chim. Acta.

[168] Kazemi F., Kiasat A.R., Sayyahi S. (2004). Chemoselective reduction of azides with sodium sulfide hydrate under solvent free conditions. Phosphorus Sulfur.

[169] Lippert A.R., New E.J., Chang C.J. (2011). Reaction-based fluorescent probes for selective imaging of hydrogen sulfide in living cells. J. Am. Chem. Soc..

[170] Peng H., Cheng Y., Dai C., Wang B. (2011). A fluorescent probe for fast and quantitative detection of hydrogen sulfide in blood. Angew. Chem. Int. Ed..

[171] Xuan W.M., Sheng C.Q., Cao Y.T., He W.H., Wang W. (2012). Fluorescent probes for the detection of hydrogen sulfide in biological systems. Angew. Chem. Int. Ed..

[172] Montoya L.A., Pluth M.D. (2012). Selective turn-on fluorescent probes for imaging hydrogen sulfide in living cells. Chem. Commun..

[173] Yu F., Li P., Song P., Wang B., Zhao J., Han K. (2012). An ICT-based strategy to a colorimetric and ratiometric fluorescence probe for hydrogen sulfide in living cells. Chem. Commun..

[174] Chen S., Chen Z.-j., Ren W., Ai H.-w. (2012). Reaction-based genetically encoded fluorescent hydrogen sulfide sensors. J. Am. Chem. Soc..

[175] Das S.K., Lim C.S., Yang S.Y., Han J.H., Cho B.R. (2012). A small molecule two-photon probe for hydrogen sulfide in live tissues. Chem. Commun..

[176] Wu Z.S., Li Z., Yang L., Han J.H., Han S.F. (2012). Fluorogenic detection of hydrogen sulfide via reductive unmasking of o-azidomethylbenzoyl-coumarin conjugate. Chem. Commun..

[177] Li W., Sun W., Yu X., Du L., Li M. (2012). Coumarin-based Fluorescent Probes for H2S Detection. J. Fluoresc..

[178] Chang S.K., Choi M.G., Cha S., Lee H., Jeon H.L. (2009). Sulfide-selective chemosignaling by a Cu(2+) complex of dipicolylamine appended fluorescein. Chem. Commun..

[179] Sasakura K., Hanaoka K., Shibuya N., Mikami Y., Kimura Y., Komatsu T., Ueno T., Terai T., Kimura H., Nagano T. (2011). Development of a highly selective fluorescence probe for hydrogen sulfide. J. Am. Chem. Soc..

[180] Hou F., Cheng J., Xi P., Chen F., Huang L., Xie G., Shi Y., Liu H., Bai D., Zeng Z. (2012). Recognition of copper and hydrogen sulfide *in vitro* using a fluorescein derivative indicator. Dalton Trans..

[181] Strianese M., Palm G.J., Milione S., Kuhl O., Hinrichs W., Pellecchia C. (2012). A FRET enzyme-based probe for monitoring hydrogen sulfide. Inorg. Chem..

[182] Hou F.P., Huang L., Xi P.X., Cheng J., Zhao X.F., Xie G.Q., Shi Y.J., Cheng F.J., Yao X.J., Bai D.C., Zeng Z.Z. (2012). A Retrievable and highly selective fluorescent probe for monitoring sulfide and imaging in living cells. Inorg. Chem..

[183] Wang M.Q., Li K., Hou J.T., Wu M.Y., Huang Z., Yu X.Q. (2012). BINOL-based fluorescent sensor for recognition of Cu(II) and sulfide anion in water. J. Org. Chem..

[184] Briglin S.M., Gao T., Lewis N.S. (2004). Detection of organic mercaptan vapors using thin films of alkylamine-passivated gold nanocrystals. Langmuir.

